# *Arabidopsis* Defense against the Pathogenic Fungus *Drechslera gigantea* Is Dependent on the Integrity of the Unfolded Protein Response

**DOI:** 10.3390/biom11020240

**Published:** 2021-02-08

**Authors:** Simone Samperna, Angela Boari, Maurizio Vurro, Anna Maria Salzano, Pierluigi Reveglia, Antonio Evidente, Angelo Gismondi, Antonella Canini, Andrea Scaloni, Mauro Marra

**Affiliations:** 1 Department of Biology, University of Rome Tor Vergata, 00133 Rome, Italy; simone.samperna@libero.it (S.S.); gismondi@scienze.uniroma2.it (A.G.); canini@uniroma2.it (A.C.); 2 Institute of Sciences of Food Production, National Research Institute, 70126 Bari, Italy; angela.boari@ispa.cnr.it (A.B.); maurizio.vurro@ispa.cnr.it (M.V.); 3 Proteomics and Mass Spectrometry Laboratory, ISPAAM, National Research Council, 80147 Naples, Italy; annamaria.salzano@cnr.it (A.M.S.); andrea.scaloni@ispaam.cnr.it (A.S.); 4 Department of Chemical Sciences, University of Naples “Federico II”, 80126 Naples, Italy; pierluigi.reveglia@gmail.com (P.R.); evidente@unina.it (A.E.)

**Keywords:** *Arabidopsis*, *Drechslera gigantea*, UPR, IRE1/bZIP60 pathway, plant immunity, salycilic acid, proteomics, microRNAs

## Abstract

*Drechslera gigantea* Heald & Wolf is a worldwide-spread necrotrophic fungus closely related to the *Bipolaris* genus, well-known because many member species provoke severe diseases in cereal crops and studied because they produce sesterpenoid phytoxins named ophiobolins which possess interesting biological properties. The unfolded protein response (UPR) is a conserved mechanism protecting eukaryotic cells from the accumulation of unfolded/misfolded proteins in the endoplasmic reticulum (ER). In plants, consolidated evidence supports the role of UPR in the tolerance to abiotic stress, whereas much less information is available concerning the induction of ER stress by pathogen infection and consequent UPR elicitation as part of the defense response. In this study, the infection process of *D. gigantea* in *Arabidopsis thaliana* wild type and UPR-defective *bzip28 bzip60* double mutant plants was comparatively investigated, with the aim to address the role of UPR in the expression of resistance to the fungal pathogen. The results of confocal microscopy, as well as of qRT-PCR transcript level analysis of UPR genes, proteomics, microRNAs expression profile and HPLC-based hormone analyses demonstrated that ophiobolin produced by the fungus during infection compromised ER integrity and that impairment of the IRE1/bZIP60 pathway of UPR hampered the full expression of resistance, thereby enhancing plant susceptibility to the pathogen.

## 1. Introduction

The endoplasmic reticulum (ER) is a pivotal eukaryotic organelle ensuring a proper folding of de novo synthesized proteins [1]. Newly assembled, unfolded polypeptides enter in the ER lumen, where they interact with enzymes ensuring N-linked glycosylation, disulfide bond formation and folding [2]. Proteins not properly folded are detected by the ER quality control machinery (ER-QC) and degraded by the ATP-dependent ubiquitin-proteasome system, according to the ER-associated protein degradation mechanism [3]. Factors affecting ER functionality bring about the accumulation of misfolded proteins in the lumen, generating ER stress that, if prolonged, can eventually lead to cell death. Eukaryotic cells restore the ER homeostasis by increasing the abundance of molecular chaperones and by enhancing ER-associated protein degradation, according to a mechanism known as unfolded protein response (UPR) [4,5].

In plants, ER stress is perceived by two ER membrane-resident proteins, i.e., the type I transmembrane protein kinase/endoribonuclease inositol-requiring enzyme 1 (IRE1) and the type II transmembrane basic leucine-zipper (bZIP) domain-containing activating transcription factor 6 (ATF6) [6]. In *Arabidopsis thaliana*, two redundant IRE1 (AtIRE1a and AtIRE1b) and three bZIP transcription factors (AtbZIP17, AtbZIP28 and AtbZIP60) were identified [7,8,9,10,11]. The IRE1-mediated unconventional splicing of bZIP60 and the regulated intramembrane proteolysis of bZIP17 and bZIP28 are the primary effectors of UPR. In physiological conditions, the IRE1 stress sensor domain binds to BiPs, while bZIP60 is anchored to the ER membrane. Upon ER stress, accumulation of misfolded proteins dissociates BiPs from IRE1, which, in turn, oligomerizes, transphosphorylates and rearranges its conformation [12], activating its RNase domain. The bZIP60 mRNA is then recruited to the basic linker region of IRE1 and unconventionally spliced [13,14]. Splicing eliminates the transmembrane domain anchoring bZIP60 to ER and yields an active transcription factor that translocates into the nucleus. Here, in association with other components, it binds to the promoter region of responsive genes eventually activating the UPR [15,16]. bZIP28 and bZIP17 are transcription factors located in the ER membrane. Upon ER stress, they are translocated into the Golgi where S1P and S2P proteases sequentially remove their transmembrane domains. The resulting active transcription factors migrate into the nucleus and promote transcription of UPR genes [10,17].

Whereas today many pieces of evidence support the involvement of UPR in the response of plants to environmental stress [18,19], information about its role in biotic stress is very poor. Components of the plant immunity system, such as receptors, signaling intermediates or antimicrobial proteins, are processed in the ER before their delivery to membranes or secretion into the apoplast. The synthesis of components of the ER-QC system was shown to be induced by pathogen perception, thereby increasing the capacity of ER to processing defensive proteins [20], whereas ER-QC mutants showed retention into ER and/or degradation of plant immunity elements [21]. Elicitation of the UPR pathway upon viral challenge has been demonstrated for potato virus x infection of *Nicotiana benthamiana* [22,23] as well as in the defense response of *A. thaliana* and *N. benthamiana* to potyviruses and potexviruses [24]. Information about the involvement of UPR in the resistance to bacterial and fungal pathogens is still very scarce. It was shown that infection by *Pseudomonas syringae* or SA treatment induced in *Arabidopsis* the transcription of AtIRE1a and AtIRE1b, whereas the *ire1a* mutant showed a greater susceptibility to the pathogen and a reduced SAR [25]. It was demonstrated that the hemibiotrophic fungus *Piriformospora indica* induces ER-stress in *Arabidopsis* during the necrotrophic stage of infection, and that the pathogen inhibits the plant UPR [26]. More recently, *Alternaria alternata* inoculation in *N. attenuata* was shown to be associated with increased levels of UPR components [27], whereas silencing of IRE1 or bZIP60 genes led to an enhanced susceptibility to the fungus. Moreover, JA-deficient or JA-insensitive mutant plants were more susceptible to the pathogen and had reduced levels of components of the IRE1/bZIP60 pathway, as well as of UPR chaperones. 

*Drechslera gigantea* Heald & Wolf is a broad-spectrum necrotrophic fungus found throughout many regions of the world [28], which infects mono-, dicots- plant weeds [29]. *D. gigantea* is closely related to the *Bipolaris* genus, which is widely studied because many species belonging to it are responsible for severe diseases of cereal crops. These species are also studied for the production of phytotoxic secondary metabolites often involved in infection processes, among which sesterterpenoid ophiobolins are well-known for their interesting biological properties [30,31].

The fungus causes a zonate eye spot disease of grasses, banana and coconut [32,33]. The molecular mechanism of *D. gigantea* infection has not been studied in depth, and collected data mostly regard phenotypic symptoms of infection [34]. Conidia cause eye spot lesions, which in turn evolve and fuse to produce leaf blight and tissue maceration [35,36,37]. Considering the capability of *D. gigantea* to infect *Arabidopsis* plants, the related type of symptoms (necrotic lesions of leaves), the concomitant production in vitro of ophiobolin A (OP-A) as the main toxin and the capability of this compound to produce necrotic symptoms resembling those caused by the fungus, this pathogen–host system was chosen to investigate the involvement of UPR in the defense response against necrotrophic fungi. This was accomplished by investigating the characteristics of infection in *bzip28 bzip60 Arabidopsis* mutant plants, where the UPR was severely hampered, with respect to wild type plants. The results of comparative qRT-PCR transcript analysis of UPR genes, proteomics, miRNAs analysis and HPLC-based hormone analysis demonstrated that UPR impairment enhanced *Arabidopsis* susceptibility to the pathogen. On the other hand, analysis of OP-A produced in vivo by the fungus during infection, as well as confocal microscopy analysis of OP-A effects in infected leaves, indicated that UPR elicitation was very likely due to the disruption of ER membrane by the phytotoxin.

## 2. Materials and Methods

### 2.1. Plant and Fungal Material, Growth Conditions, OP-A production, Plant Inoculation and Treatments

For in-soil plant growth, 10 mg of seeds from wild type (WT), ecotype Columbia 0 (Col-0) and from *bzip28 bzip60* mutant (provided by Prof. Federica Brandizzi, MSU-DOE Plant Research Laboratory, Michigan State University, USA; Salk numbers: *bzip28*, Col-0; SALK_132285, *bzip60-1*, Col-0; SALK_050203) *A. thaliana* plants, or from GFP-tmKKXX-expressing *A. thaliana* plants (provided by Prof. P. Schafer, School of Life Sciences, Warwick University, Coventry, UK), were added to 1 mL of deionized water and kept at 4 °C for 4 days, in the dark. After stratification, seeds were suspended in 0.1% agarose and scattered in pots filled with universal soil. Pots were placed in a climatic chamber (VB1514 Vötsch, Balingen, Germany), at 22 °C, at 80% humidity, with a 16/8 h light/dark cycle, and kept growing for 3 weeks. An isolate of *D. gigantea* Head & Wolf (strain ITEM 7004, from the ISPA-CNR collection, Bari, Italy) was grown on potato dextrose agar (PDA) medium, in climatic chamber, at 25 °C, in the dark. It was transferred to fresh medium once a week, through a suspension of mycelia in deionized water. For infection of *A. thaliana* leaves, fungal conidia suspensions were obtained by filtration of mycelia through a sterile gauze, and the quantity of conidia was estimated by direct counting on a Thoma chamber. *D. gigantea* infections were carried out on soil-grown *A. thaliana* WT or *bzip28 bzip60* mutant plants, as well as on *A. thaliana* GFP-tmKKXX-expressing plants. Three-week-old plants were uniformly sprayed until a complete wetting with fungal suspension containing 500,000 conidia/mL in deionized water, containing 0.05% *w/v* Tween 20; mock plants were treated with the same solution not containing *D. gigantea* conidia. To create a high-humidity environment favorable for fungal growth, plants were then covered with transparent boxes. For in vitro treatments with OP-A, leaves from three-week-old wild type *A*. *thaliana* plants were detached and submerged in 10 mM Tris/Mes buffer pH 6.5, containing 20 µM OP-A. The samples were vacuum infiltrated for 10 min, and then incubated for different times. OP-A was obtained as white crystals by extraction and purification of *D. gigantea* culture filtrates, as reported by Evidente et.al [36].

### 2.2. qRT-PCR Analysis of Genes and MicroRNAs Expression

For qRT-PCR analysis of gene expression, WT and *bzip28 bzip60* mutant *Arabidopsis* plants inoculated with *D. gigantea* as reported above were subjected to harvesting of leaves 6 or 24 h after fungal treatment; the same was done for mock plants. Total RNA was extracted from homogenized leaves using RiboZOL (vWR, Radnor, PA, USA). For cDNA synthesis, 20 μg of total RNA was retro-transcribed by using the FastGene Scriptase II cDNA kit (NIPPON Genetics EUROPE), according to the manufacturer’s instructions, and stored at −80 °C until use. qRT-PCR experiments were performed according to [38], using the LightCycler apparatus (Roche, Basel, Switzerland) and the SYBR GREEN dye (PCR Biosystems, London, UK). The 2^-ΔΔCt^ method was applied to evaluate the level of gene expression, using actin-8 *Arabidopsis* gene (ACT8) as housekeeping gene. The results represent mean values ± SD of independent experiments (*n* = 3). Samples were run in technical triplicates. Statistical significance was attributed by Student’s test (*p* < 0.05). The primers used for amplification are listed in Table S1. For microRNA expression analysis, microRNAs (miRNAs) were purified from the leaf tissues with mirPremier microRNA Isolation kit (Sigma-Aldrich) and retro-transcribed with the miRCURY LNA Universal RT microRNA PCR Synthesis kit II (EXIQON) [39]. Then, qPCR amplifications were performed using microRNA LNA PCR primer sets (EXIQON) in a Biorad (IQ5) thermocycler. MicroRNA quantifications was performed by the 2^-ΔΔCt^ method, using 5S rRNA (GenBank ID: AB073495.1) as internal loading control. Values represent the mean values ± SD of independent measurements (*n* = 4). Statistical significance was attributed by one-way ANOVA test (* *p* < 0.05, ** *p* < 0.01). The list of primers used for amplification is reported in Table S2.

### 2.3. Total Chlorophyll Assay

One hundred milligrams of leaves from WT or *bzip28 bzip60* mutant *Arabidopsis* plants inoculated with *D. gigantea* as reported above, or from mock plants, were collected in a Falcon tube with 5 mL of DMSO (Panreac). After incubation at 65 °C for 90 min, and subsequent cooling at 25 °C, the supernatants were collected and total chlorophyll (Chl) content was estimated with a spectrophotometer (SmartSpec 3000, Bio-Rad, Hercules, CA, USA), by measuring absorption at 663 and 645 nm. Chl quantification (g/L) was performed according to [40].

### 2.4. Membrane-Lipid Peroxidation Assay

Membrane-lipid peroxidation was estimated by the malondialdehyde (MDA) method [41]. One hundred milligrams of leaves from WT or *bzip28 bzip60* mutant *Arabidopsis* plants inoculated with *D. gigantea* as reported above, or from mock plants, were homogenized in liquid N_2_, suspended in 500 µL of 0.1% trichloroacetic acid (TCA) and centrifuged at 15,000× *g* for 10 min, at 4 °C. One hundred microliters of the supernatant were added to 1.5 mL of 0.5% thiobarbituric acid in 20% TCA and incubated for 25 min, at 95 °C. After incubation, the reaction was blocked by placing the samples in ice. After cooling at 25 °C, sample absorbance was measured at 532 nm and 600 nm. The absorbance values measured at 600 nm were subtracted from those measured at 532 nm, and MDA concentration values were calculated by interpolation with a calibration curve obtained with known amounts of MDA. 

### 2.5. Electrolyte Leakage Assay

Two hundred milligrams of leaves from WT or *bzip28 bzip60* mutant *Arabidopsis* plants inoculated with *D. gigantea* as reported above, or from mock plants, were cut into 5 mm strips and submerged in 30 mL of deionized water, for 2 h, at 25 °C. After incubation, the electrical conductivity was measured by a conductimeter (AD31, Adwa, Szeged, Hungary) and relative electrical conductivity (REC %) values calculated. Boiled samples were used to determine maximum percentage of electrolyte leakage, which was then calculated using the following formula:

REC % = C1/C2 × 100 (1)

where C1 is the conductivity at 25 °C and C2 is the conductivity at 100 °C.

### 2.6. In Situ H_2_O_2_ Production Assay

H_2_O_2_ production in leaves was detected by staining with 3,3′-diaminobenzidine tetrahydrochloride (DAB), according to [42]. Five leaves from WT or *bzip28 bzip60* mutant *Arabidopsis* plants inoculated with *D. gigantea* as reported above, or from mock plants, were submerged in a solution of 10 mM DAB, pH 6.8, containing 0.05% *w/v* Tween 20. After vacuum infiltration for 15 min and incubation for 5 min under stirring, leaves were submerged in a bleaching solution containing ethanol:acetic acid:glycerol (3:1:1 v/v/v) and boiled for 15 min to remove chlorophyll. After cooling at 25 °C, the bleaching solution was eliminated, fresh bleaching solution added and leaves mounted on glass slides for observation with an optical/epifluorescent microscope (Wilovert S, Helmut Hund GmbH, Wetzlar, Germany).

### 2.7. Callose Deposition Assay

Leaves from WT or *bzip28 bzip60* mutant *Arabidopsis* plants inoculated with *D. gigantea* as reported above, or from mock plants, were collected and placed in Eppendorf tubes. Chlorophyll was removed by incubation in a solution of acetic acid:ethanol (1:3 *v/v*), overnight, at 25 °C. After incubation, the solution was removed and replaced with a solution of 150 mM K_2_HPO_4_, pH 6.8, for 30 min. Leaves were then submerged in a solution of 0.01% *w/v* aniline blue in 150 mM K_2_HPO_4_, pH 6.8, for 2 h. After incubation, leaves were treated with 50% *v/v* glycerol and mounted on glass slides for observation with an optical/epifluorescent microscope (ECLIPSE TE2000-E, Nikon, Melville, New York, NY, USA).

### 2.8. HPLC Analysis of Salicylic and Ophiobolin

For the extraction of salicylic acid (SA) from WT or *bzip28 bzip60* mutant *Arabidopsis* plants inoculated with *D. gigantea* as reported above, or from mock plants, a modified version of the method of pH switch was used [43] The HPLC system used for the analysis of hormone extracts was from Hitachi (Chijoda, Tokyo, Japan), and consisted of a pump (model 5160) and a spectrophotometric detector (model 5410). Metabolite separation was performed using a Phenomenex Luna (Darmstadt, Germany) C18 reversed-phase column (15 × 4.6 mm; 5 μm particle size), which was eluted at a flow rate of 1 mL/min. The gradient started from 5% ACN, linearly increased to 40% ACN in 13 min, remained in isocratic condition for 2 min, then linearly increased to 80% ACN in 30 min, and finally followed by a re-equilibrium phase at initial gradient composition for 5 min. Detection was performed at 244 nm for SA and 228 nm for JA. Separated compounds were identified through their retention times, UV spectra and relative literature data, by comparison with pure SA and JA standards (Sigma-Aldrich, St. Louis, MO, USA). These standard compounds were also used to build up calibration curves (in the range 5–200 μg/mL). For quantitative analysis, two different amounts from WT and mutant samples were injected in triplicate. The reported values represent the concentration (expressed as μg of hormone/g of fresh tissues). The mean value ± SD of three independent extractions is provided. For the extraction of OP-A from leaves of WT plants infected by *D. gigantea*, 3 g of three-week-old leaves were collected and grinded in liquid N_2_ with mortar and pestle. The resulting powder was suspended in 1 mL methanol. The extract was cleared by centrifugation at 16,000× *g*, for 10 min, at 4 °C. After concentration under vacuum to 100 µL, the methanolic extract was stored at–80° C until final HPLC analysis.

### 2.9. Protein Extraction, Digestion and Peptide Fractionation

One gram of leaves from WT or *bzip28 bzip60* mutant *Arabidopsis* plants inoculated with *D. gigantea* as reported above, or from mock plants, was collected and grinded in liquid N_2_ with mortar and pestle. The resulting powder was suspended in 2.5 mL of methanol, and left in incubation at 4 °C, overnight. After incubation, the sample was centrifuged at 2000× *g*, for 10 min, at 4 °C, and the supernatant was removed. The pellet was washed three times with ice-cold acetone and dried in a SpeedVac vacuum concentrator (ThermoFisher Scientific, Waltham, MA, USA). The dried pellet was stored at −20 °C until used. Protein pellets (25 mg) were resuspended in 750 µL of 8 M urea, 50 mM triethylammonium bicarbonate (TEAB), pH 8.5, supplemented with a protease inhibitors cocktail for plant tissues (Sigma-Aldrich, St. Louis, MI, USA) and 1 mM phenylmethanesulfonyl fluoride (PMSF). Suspensions were thoroughly mixed, incubated at 30, °C for 1 h, and finally sonicated (50 W output), for 10 s, twice, with a 60 s rest. Samples were centrifuged at 12,000× rpm, for 1 h, at 4 °C, and the protein concentration was determined on the supernatants using the Pierce BC Protein assay kit™ (Thermo Scientific, Rockford, IL, USA). An aliquot of each protein sample (100 μg) was adjusted to 100 µL with 100 mM TEAB, and then separately treated with the TMT10plex™ Isobaric Label Reagent (Thermo-Fisher Scientific, Waltham, MA, USA), following the instructions of mass tagging for protein reduction, alkylation, digestion and labeling, as already described [44] Three independent replicates were labeled according to the scheme: WTContr for TMT6-128N, WTinfected for TMT6-129N, *bzip28 bzip60* Contr for TMT6-126 and *bzip28 bzip60* infected for TMT6-127N, at 25 °C, according to manufacturer’s instructions. The labeling reaction was quenched by adding 8 µL of 5% *w/v* hydroxylamine to the mixture, for 15 min. An aliquot of each peptide mixture was mixed in equal molar ratios and dried under vacuum. The pooled TMT-labeled peptide mixture was dissolved in 0.1% formic acid (FA) and fractionated using the Pierce™ High pH Reversed-Phase Peptide fractionation kit (Thermo-Fisher Scientific), according to manufacturer’s instructions. The eight fractions eluted from the High pH-RP column were vacuum-dried and reconstituted in 0.1% FA for mass spectrometric analysis.

### 2.10. Mass Spectrometry and Data Analysis 

NanoLC-ESI-Q-Orbitrap-MS/MS analysis was carried out on an UltiMate 3000 HPLC RSLC nano system (Dionex, Sunnyvale, CA, USA) interfaced to a Q-ExactivePlus mass spectrometer through a Nanoflex ion source (Thermo-Fisher Scientific, Waltham, MA, USA). Peptides were loaded on an Acclaim PepMapTM RSLC C18 column (150 mm × 75 μm, 2 μm particles, 100 Å pore size) (Thermo-Fisher Scientific) and eluted with a gradient of solvent B (80% ACN/20% water, 0.1% FA) in solvent A (acqueous 0.1% FA), at a flow rate of 300 nL/min. For gradient elution, solvent B was increased from 5% to 60% in 125 min and from 60% to 95% over 1 min. Full scan spectra were acquired in the *m/z* range 375-1500, at nominal resolution of 70,000. Data-dependent acquisition was performed on the 15 most abundant ions, using a dynamic exclusion duration value of 30 s. MS^2^ spectra were acquired at a resolution of 17,500, using an isolation width of *m/z* 1.2, and a normalized collision energy of 32%. Automatic gain control target was set at 10^5^ and the maximum ion target at 120 ms. NanoLC-ESI-Q-Orbitrap-MS/MS analysis of the eight peptide fractions was performed in technical triplicate. Raw data were analyzed for protein identification and relative quantification by Proteome Discoverer suite v2.4 (Thermo Scientific), using Mascot v2.6.1 (Matrix Science, UK) as searching algorithm against the TAIR10 database (www.arabidopsis.org, July 2019) of *A. thaliana* protein sequences and the most common protein contaminants. Cys-carbamidomethylation and TMT6plex modification of peptide N-terminal and Lys were selected as fixed modifications, while Met-oxidation, Asn/Gln deamidation and pyroglutamate formation at Gln were set as variable modifications. Two missed cleavages were chosen as maximum value for trypsin proteolysis. Peptide identification data were filtered using a Mascot ion score threshold ≥30, and at least two sequenced peptides were required to confirm protein identification. Only high-confidence protein identifications (corresponding to FDR 1%) were retained. Protein abundance values were obtained from TMT reporter ion intensities in the MS/MS spectra from raw datasets. Results were filtered to retain only significant data showing an abundance ratio *p*-value < 0.05; after this preliminary step, quantitative abundance differences for individual proteins were evaluated. Final proteomic data were deposited to the ProteomeXchange consortium via the PRIDE partner repository with the dataset identifier PXD018099. Functional analysis of the differentially represented proteins (DRPs) was performed as done previously [45]. It was obtained with a preliminary Mercator software analysis [46], which was further integrated with information from the Bevan classification [47] and recent literature data.

### 2.11. Confocal Microscopy

Confocal microscopy experiments were conducted on leaves from *A. thaliana* GFP-tmKKXX-expressing plants, which were infected with a suspension of 500,000 conidia/mL of *D*. *gigantea* for 6 and 24 h, or treated with OP-A, as reported above. Images were acquired with a FV1000 Olympus (Hamburg, Germany) laser scanning confocal microscope, with an argon 488 nm laser for GFP excitation and 635 nm for chlorophyll excitation, using a 60× oil objective (N.A.: 1,35) in z stack mode (step size 0.45 µM). Images were processed by Imaris software (Bitplane, Zürich, Switzerland).

## 3. Results

### 3.1. D. gigantea Induced Larger Necrotic Lesions and Higher Chlorophyll Loss in bzip28 bzip60 Mutant Plants 

**Figure 1:**
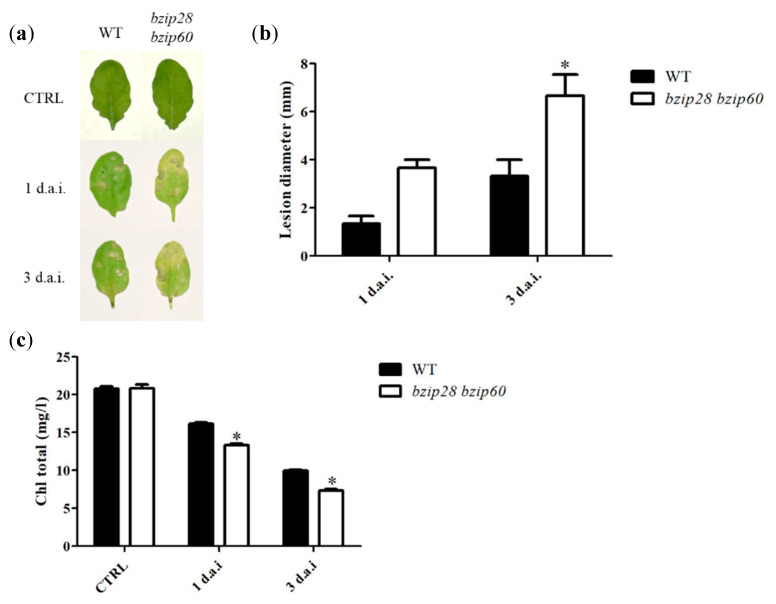
Phenotype (**a**), necrotic areas (**b**) and chlorophyll content (**c**) of WT and *bzip28 bzip60* mutant *Arabidopsis* plants infected by *D. gigantea.* (**a**) WT and *bzip28 bzip60* mutant plants were sprayed with a suspension of *D. gigantea* conidia (500,000 /mL). Leaves were collected 1 and 3 d.a.i. and subjected to optical image recording. (**b**) Diameter of necrotic lesions was measured after 1 and 3 d.a.i. on detached leaves inoculated with 5-µL droplets of fungal conidia suspension. The results represent mean values ± SD (*n* > 10). Statistical significance was attributed by Student’s test (* *p* < 0.05). (**c**) Leaves from WT and *bzip28 bzip60* mutant plants treated as reported in (**a**) were extracted for determination of total chlorophyll, and the corresponding values determined (mg/L) as described in Section 2.3. The results represent mean values ± SD (*n* > 10). Statistical significance was attributed by Student’s test (* *p* < 0.05).

The progression of fungal infection was comparatively evaluated as development of necrotic areas and chlorophyll bleaching in three-week-old WT and *bzip28 bzip60* mutant plants. Before inoculation, WT and mutant plants showed an indistinguishable phenotype. One day after pathogen inoculation (1 d.a.i), brown spots were clearly visible on leaves, and 3 d.a.i. the number and size of necrotic areas resulted increased in both WT and mutant plants (Figure 1a). A quantitative comparison was performed by inoculating detached leaves with 5-µL droplets of fungal conidia suspension and measuring the diameter of necrotic areas. The average diameter of necrotic spots was larger in the *bzip28 bzip60* mutant than in WT leaves, at both 1 and 3 d.a.i (Figure 1b). As shown in Figure 1c, fungal infection also caused a reduction the chlorophyll content of the leaves, which was detectable already 1 d.a.i. and that resulted further decreased 3 d.a.i.; this decrease was greater in the *bzip28 bzip60* mutant than in WT plants.

### 3.2. D. gigantea Induced Higher Ion Leakage and Membrane Lipid Peroxidation in bzip28 bzip60 Mutant Plants

**Figure 2:**
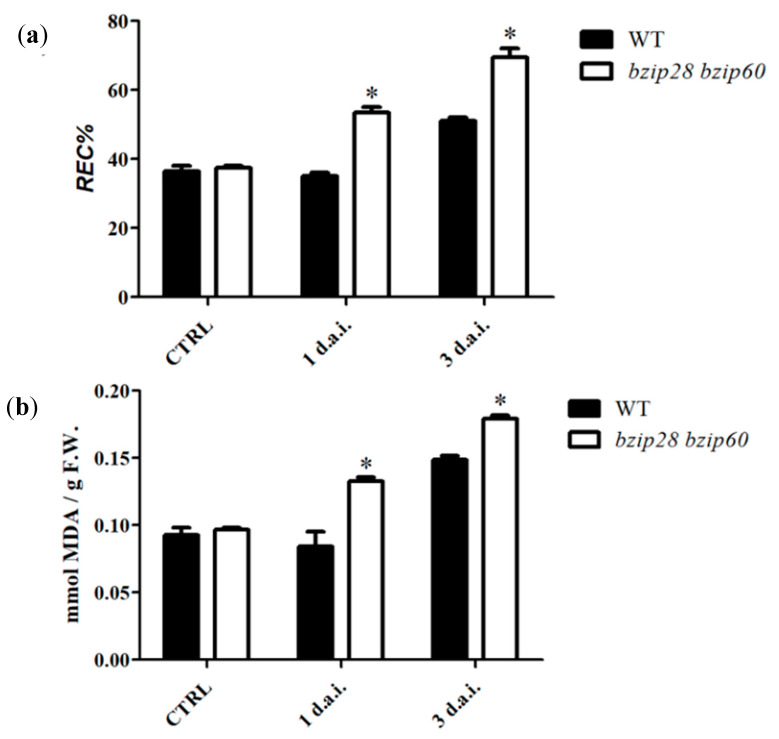
Ion leakage (**a**), and lipid peroxidation (**b**) of leaves from WT and *bzip28 bzip60 Arabidopsis* plants infected by *D. gigantea*. Leaves of WT and *bzip28 bzip60* mutant plants were sprayed with a suspension of *D. gigantea* conidia (500,000 /mL) and then collected 1 and 3 d.a.i. Ion leakage and membrane lipid peroxidation were determined as REC% and MDA content, respectively, as reported in Section 2.5 and Section 2.4. The results represent mean values ± SD (*n* > 10). Statistical significance was attributed by Student’s test (* *p* < 0.05).

To evaluate the degree of cell injury caused by *D. gigantea* infection, ion leakage and lipid peroxidation assays were performed on leaves of WT and *bzip28 bzip60* mutant plants. Measurements showed that values of relative electric conductivity (REC%) increased in all samples from 1 to 3 d.a.i. (Figure 2a), indicating that the necrotrophic infection provoked cell membrane damage. In particular, REC % values of the *bzip28 bzip60* mutant were higher than those of WT, at both 1 and 3 d.a.i., suggesting that infection of *D. gigantea* proceeded faster and was more harmful to plasma membrane in the *bzip28 bzip60* mutant than in WT. Lipid peroxidation assay showed that MDA levels increased in all samples from 1 to 3 d.a.i, and that they were higher in *bzip28 bzip60* mutant plants as compared to WT (Figure 2b). The latter was in accordance with ion leakage data and confirmed that during infection the pathogen caused an increasing membrane damage, which was more pronounced in the *bzip28 bzip60* mutant as compared to WT.

### 3.3. D. gigantea Induced Higher Hydrogen Peroxide Production in bzip28 bzip60 Mutant Plants

**Figure 3:**
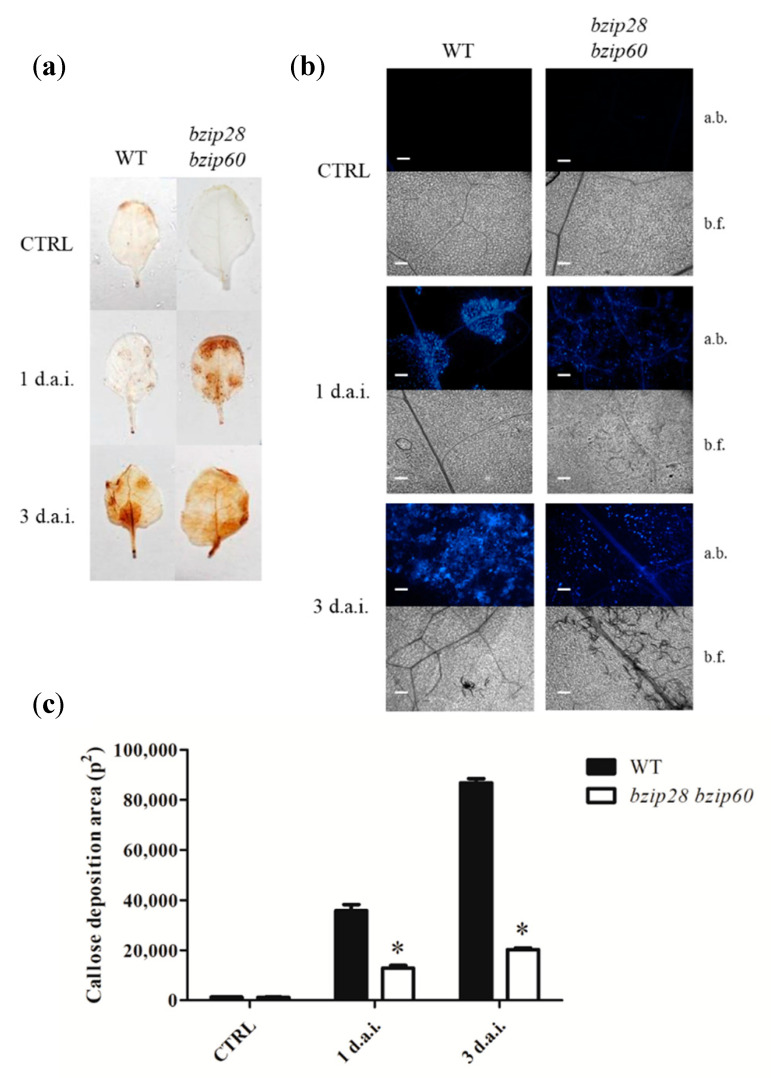
In situ H_2_O_2_ production (**a**), and callose deposition (**b**,**c**) in leaves of WT and *bzip28 bzip60* mutant *Arabidopsis* plants infected with *D. gigantea*. (**a**) Optical microscope images of leaves of WT and *bzip28 bzip60* mutant plants. Leaves were collected 1 and 3 d.a.i., stained with DAB as reported in Section 2.6 and observed using an optical microscope at 4× magnification; bar = 200 μm. Images are representative of three independent experiments. (**b**) Fluorescence microscope images of leaves from WT and *bzip28 bzip60* mutant plants sprayed with a suspension of *D. gigantea* conidia (500,000/mL). The leaves were collected 1 and 3 d.a.i., stained with 0.01% aniline blue and observed using a fluorescence microscope at 10× magnification as reported in Section 2.7. Images are representative of three independent experiments; a.b., aniline blue; b.f., bright field. (**c**) Callose deposition area as derived from images reported in (**b**), following analysis with ImageJ software. The results represent mean values ± SDs (*n* > 10). Statistical significance was attributed by Student’s test (* *p* < 0.05).

ROS production is a marker of basal defense activation upon pathogen attack. Among the different ROS species produced by plants, stable hydrogen peroxide (H_2_O_2_) can be in situ detected after its reaction with 3,3′-diaminobenzidine (DAB) cromogen, catalyzed by endogenous peroxidases [48], which determines the formation of a brownish precipitate. Figure 3a shows the DAB staining of leaves from WT and *bzip28 bzip60* mutant *Arabidopsis* plants infected with *D. gigantea*; no H_2_O_2_ production occurred in non-infected plants (CTRL), whereas DAB staining revealed H_2_O_2_ production 1 d.a.i., which was increased 3 d.a.i., in both WT and mutant leaves. Interestingly, *bzip28 bzip60* mutant leaves showed a higher H_2_O_2_ production as compared to WT at both sampling times. Since over-production of ROS is generally related to plant cell death and enhanced susceptibility to necrotrophic pathogens [49,50], overall, these data suggested a higher susceptibility of the *bzip28 bzip60* mutant to pathogen infection.

### 3.4. bzip28 bzip60 Mutant Plants Showed Reduced Callose Deposition upon D. gigantea Infection 

The deposition of callose within the cell walls in contact with the pathogen is a component of the basal defense response common to many plants, including *Arabidopsis* [51]. The results of aniline blue staining showed that the pathogen triggered callose deposition already 1 d.a.i., which was increased 3 d.a.i (Figure 3b). Callose deposition appeared considerably lower in mutant plants. Quantitative image pixel analysis (Figure 3c) confirmed that callose deposition was significantly reduced in the *bzip28 bzip60* mutant as compared to WT, thereby suggesting a partial impairment of the defensive response and corroborating other data indicating a higher susceptibility of the mutant to the fungal pathogen.

### 3.5. The IRE1/bZIP60 Pathway of UPR Was Induced in WT But Not in bzip28 bzip60 Mutant Plants upon D. gigantea Infection

**Figure 4:**
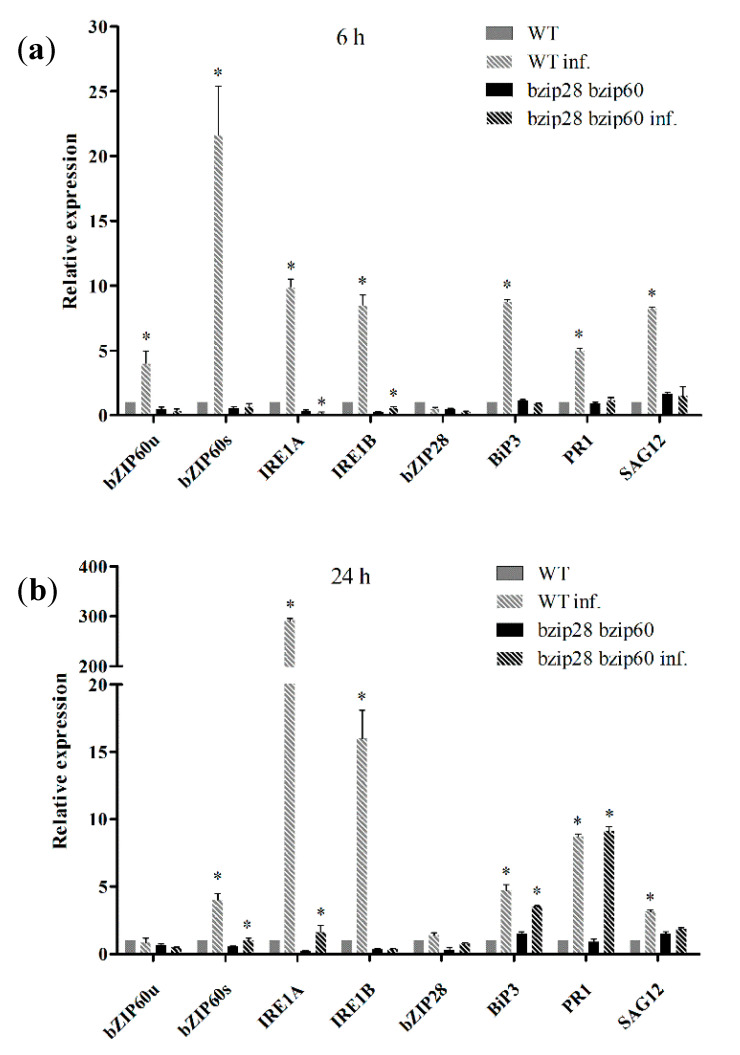
UPR genes transcription in leaves from WT and *bzip28 bzip60* mutant *Arabidopsis* plants infected by *D. gigantea*. Analysis of relative expression of IRE1a, IRE1b, bZIP60u, bZIP60s, bZIP28, BiP3, SAG12, and PR-1 genes, following inoculation of WT and *bzip28 bzip60* mutant *Arabidopsis* plants with a suspension of *D. gigantea* conidia (500,000/mL), for: 6 h (**a**), and 24 h (**b**). mRNA levels were quantified by qRT-PCR using ACT8 as housekeeping gene. The results of the infected plants are reported as fold changes with respect to non-infected WT (6 h) considered as unit. The results represent the mean values ± SD of independent experiments (*n* = 3). Samples were run in technical triplicates. Statistical significance was attributed by one-way ANOVA test (* *p* < 0.05).

Since very few data are available concerning the involvement of plant UPR in the response to infections caused by necrotrophic fungi, we tested whether inoculation of *D*. *gigantea* in WT *Arabidopsis* plants elicited transcription of UPR marker genes. The analysis was performed in comparison to *bizip28 bzip60* mutant plants to correlate UPR to the expression of resistance toward the pathogen. The results of qRT-PCR analysis show that *D. gigantea* challenge induced a significant increase of transcripts of genes of the IRE1/bZIP60 pathway of UPR in WT plants, already 6 h after inoculation (Figure 4a). In fact, levels of both isoforms (a and b) of the ER stress sensors IRE1 were consistently increased, as well as those of the bZIP60s effector, as a result of the splicing activity of IRE1 on the bZIP60 mRNA. Levels of bZIP28, the main marker of the second branch of plant UPR, were not affected. Conversely, UPR gene transcripts induction was impaired in the *bzip28 bzip60* mutant, as compared to WT plants. Twenty-four hours after inoculation, IRE1a and IRE1b levels were increased in WT plants, as compared to 6 h, whereas transcription of bZIP60s was significantly reduced. In mutant plants, the transcription of IRE1a and bZIP60s showed a very slight increase from 6 to 24 h infection (Figure 4b). The transcription of genes of downstream effectors of UPR (BiP3), and of genes of UPR-associated processes, e.g., pathogenesis related protein 1 (PR-1) (secretion of defensive proteins) and senescence associated gene 12 (SAG12) (cell death), was also monitored. The results in WT plants show that transcription of the gene of BiP3 chaperone was higher at 6 h than at 24 h, thereby paralleling the trend observed for bZIP60s; in mutant plants, induction occurred only after 24 h infection and to a reduced extent as compared to WT plants. Levels of transcripts of PR-1, the major defensive secretory protein in *Arabidopsis*, were increased 6 h after infection only in WT plants, while, after 24 h, resulted increased to the same extent both in WT and mutant plants. Transcript levels of SAG12 gene, which is associated with delayed senescence in *Arabidopsis* and negative regulation of cell death in rice, in response to pathogen infection [52], were increased only in WT plants after 6 h infection and to a lesser extent after 24 h.

Overall, qRT-PCR results provide evidence that in WT plants *D*. *gigantea* elicited a robust activation of the IRE1/bZIP60 pathway of UPR after 6 h infection. After 24 h, when cellular damage was more pronounced, the response declined considerably. On the other hand, as expected, in the *bzip28 bzip60* mutant, the UPR was constitutively impaired. Since the *bzip28 bzip60* mutant was also more susceptible to infection than WT, qRT-PCR results demonstrated that UPR impairment hampers the defense response to *D. gigantea*, and that the IRE1/bZIP60 pathway plays a significant role in the expression of resistance toward necrotrophic fungi. Concerning the second arm of the UPR pathway, the lack of induction of the bZIP28 gene in the infected WT plants was in accordance with previous work demonstrating that the IRE1/bZIP60 pathway of UPR is primarily involved in plant defense [19,25,27], whereas no data are available for the bZIP28 pathway, which appears mainly involved in abiotic stress or hormone-mediated responses [19].

### 3.6. D. gigantea Impaired the Integrity of ER During Infection 

**Figure 5:**
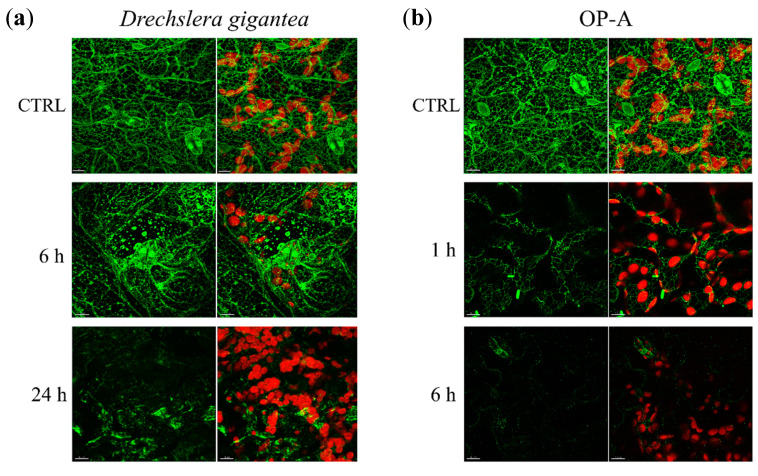
Confocal microscope imaging of ER network of *Arabidopsis* leaves: infected by *D. gigantea* (**a**) or infiltrated with OP-A (**b**). (**a**) Leaves of GFP-tmKKXX-expressing WT *Arabidopsis* plants were inoculated or not with a suspension of *D. gigantea* conidia (500,000/mL); after 6 and 24 h from infection, they were analyzed by confocal microscope at 488 nm (argon laser) for GFP excitation. (**b**) Leaves of GFP-tmKKXX-expressing WT *Arabidopsis* plants were detached from plants, incubated with 20 μM OP-A for 1 and 6 h, as reported in Section 2.11, and analyzed by confocal microscope at 488 nm (argon laser) for GFP excitation. Left, GFP fluorescence (green). Right, merged chloroplast autofluorescence (red), and GFP fluorescence (green) images. Bar = 10 μm.

To ascertain whether plant cell ER was a target of *D. gigantea* during infection, a confocal microscope analysis was performed on fungus-inoculated leaves of WT *Arabidopsis* plants expressing GFP-tmKKXX. This fluorescent protein localizes into ER, thereby visualizing its structure [53]. Recorded images shown in Figure 5a demonstrate that, as early as 6 h after infection, a partial loss of the continuous ER network and the appearance of globular ER structures occurred. After 24 h of infection, continuous ER network appeared totally disintegrated, with a predominance of globular structures, indicating extensive ER vacuolization.

It is known that ophiobolins affect membrane permeability and it has recently been shown that OP-A interacts with phoshatidylethanolamine residues, thereby inducing lipid bilayer destabilization [54]. Hence, to investigate whether the diffuse ER network destruction could be ascribed to chemical action of the phytotoxin rather than to mechanical damage of penetrating hyphae, confocal microscope analysis was performed also on *Arabidopsis* leaves infiltrated with 20 μM OP-A. The recorded images in Figure 5b, show that the ER network of infiltrated leaves was partially disrupted after 1 h incubation, and almost completely destroyed after 6 h incubation. This result prompted us to investigate the effect of 20 μM OP-A infiltration of leaves of WT plants on the elicitation of UPR. The results from qRT-PCR analysis reported in Figure S1 demonstrate that OP-A (6 h incubation) elicited the transcription of main marker genes of the IRE1/bZIP60 pathway of UPR, such as bZIP60s and BIP3. Finally, HPLC analysis of methanolic extracts of WT control and infected leaves allowed us to ascertain that OP-A was actually produced within infected leaves at amounts comparable to those provided exogenously (Figure S2). 

Overall, these results demonstrated that: (i) the ER was a primary target of fungal infection; (ii) the disaggregation of the ER network was due to the action of the OP-A produced during infection; (iii) the ER insult brought about activation of UPR.

### 3.7. D. gigantea Markedly Increased SA Concentration in WT But Not in bzip28 bzip60 Mutant Plants 

The defensive hormone salicylic acid (SA) is known to be an UPR activator. In fact, SA was reported to induce UPR genes when exogenously administered to *Arabidopsis* [55] or following pathogen infection [56]. Hence, we analyzed the SA content of leaves from WT or *bzip28 bzip60* mutant *Arabidopsis* plants not infected by *D. gigantea* or after infection for 6 and 24 h.

**Figure 6:**
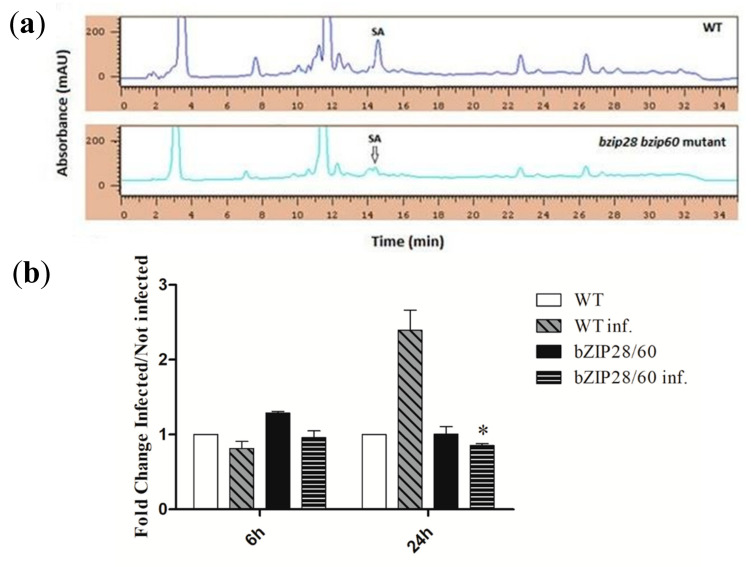
HPLC determination of SA content (**a**) and qRT-PCR analysis of ICS1 gene transcription (**b**) in leaves from WT and *bzip28 bzip60* mutant *Arabidopsis* plants infected by *D. gigantea* for 24 h. (**a**) Exemplificative chromatographic profile of a sample of extract from leaves of WT (Top) and *bzip28 bzip60* mutant (Bottom) *Arabidopsis* plants infected by *D. gigantea* for 24 h. (**b**) mRNA levels were quantified by qRT-PCR, using ACT8 as housekeeping gene. The results of the infected plants are reported as fold changes with respect to non-infected WT (6 h) considered as unit. The results represent the mean values ± SD of independent experiments (*n* = 3). Samples were run in technical triplicates. Statistical significance was attributed by Student’s test (* *p* < 0.05).

Although SA levels in leaves from both WT and mutant plants under control conditions or after 6 h infection were very close to the detection limit of our analytical system to be accurately ascertained, a significant concentration difference emerged between WT and mutant plants infected for 24 h. A typical HPLC chromatogram of extracts from WT and mutant leaves infected by the fungus for 24 h is reported in Figure 6a. SA concentration in leaves from WT plants was markedly higher as compared to that of *bzip28 bzip60* mutant leaves (estimated mean values from three independent extractions were 36.8 ± 0.8 and 8.1 ± 0.7 ng SA /g fw for WT and mutant leaves, respectively).

To confirm this result, qRT-PCR analysis of transcription of the gene encoding isochorismate synthase 1 (ICS1), a pivotal enzyme in SA synthesis and whose levels are generally increased after pathogen challenge, was performed [57]. As shown in Figure 6b, the ICS1 gene transcript was not induced after 6 h infection, whereas corresponding mRNA levels were increased in leaves from WT plants after 24 h, but not in those from the *bzip28 bzip 60* mutant. These results indicate that mutant plants, which were more susceptible to *D. gigantea*, had reduced levels of SA as compared to WT plants. Although SA biosynthesis and signaling is usually associated with the defense against biotrophic pathogens, recent gene expression studies have shown that SA can be involved also in the response to necrotrophic pathogens [58]. For example, exogenous administration of SA to tomato leaves was shown to elicit the expression of the PR1 gene and to increase plant resistance to *Botrytis cinerea*, which is a model fungus of necrotrophic interaction [59].

### 3.8. D. gigantea Elicited a More Robust Synthesis of Defense and Stress-Related Proteins in WT Than in bzip28 bzip60 Mutant Plants

**Figure 7:**
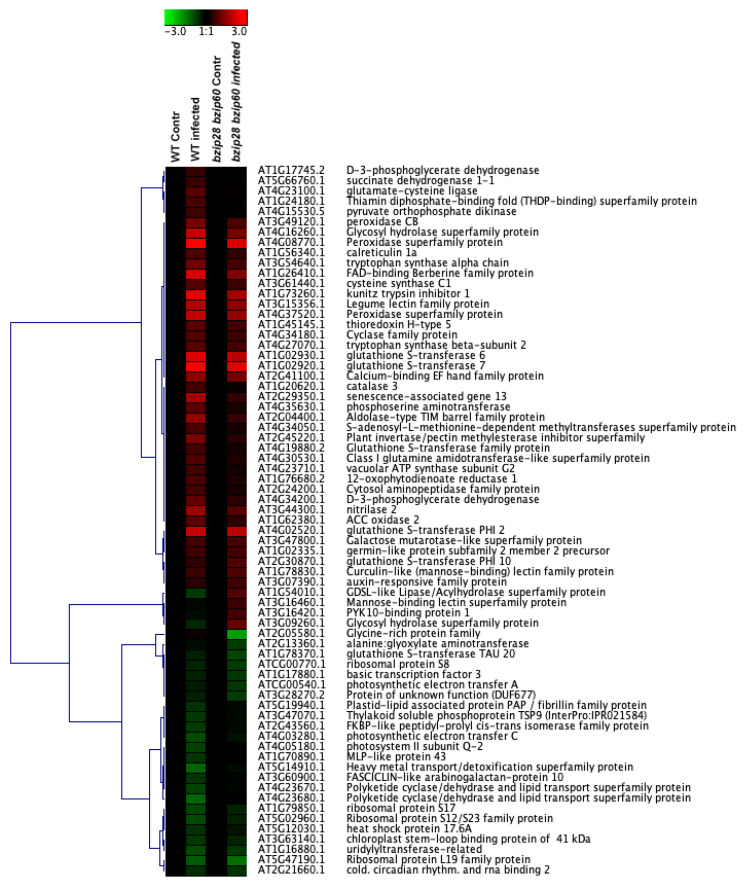
Differentially represented proteins in leaves from WT and *bzip28 bzip60* mutant *Arabidopsis* plants infected by *D. gigantea* for 24 h, as deduced by proteomic analysis. An independent pairwise comparison of leaves of infected WT vs. non-infected WT plants, as well as of infected *bzip28 bzip60* mutant vs. non-infected *bzip28 bzip60* mutant ones is shown. A heat-map representation and hierarchical clustering analysis of differentially represented proteins is given. The latter was performed using Genesis 1.8.1 platform.

**Figure 8:**
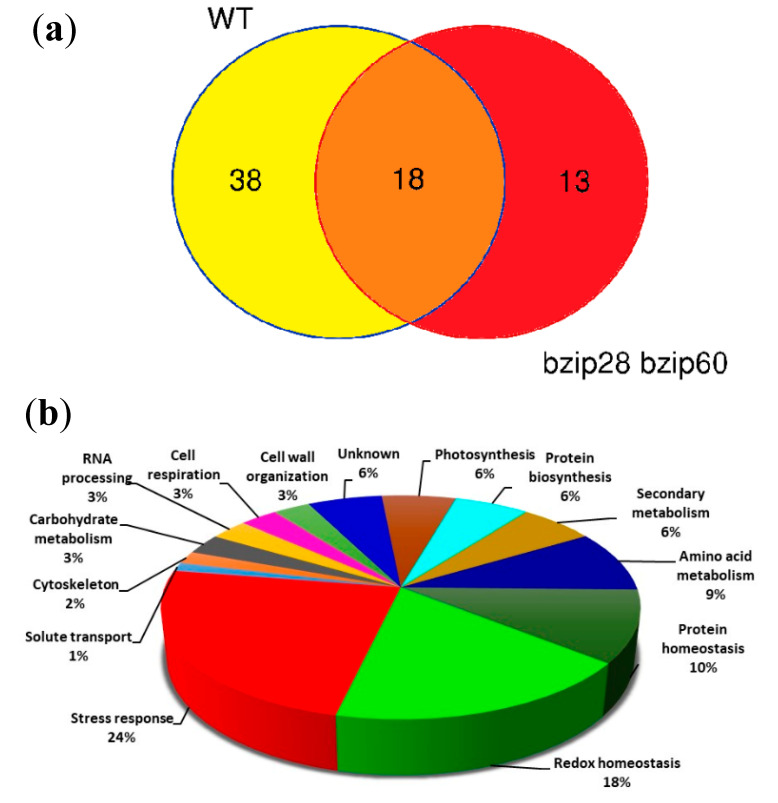
Differentially represented proteins in leaves from WT and *bzip28 bzip60* mutant *Arabidopsis* plants infected or not by *D. gigantea* for 24 h. (**a**) Venn diagram showing common and specific differentially represented proteins between WT and *bzip28 bzip60* mutant plants. (**b**) Functional classification of differentially represented proteins in leaves from WT and *bzip28 bzip60* mutant *Arabidopsis* plants. An independent pairwise comparison of leaves of infected WT vs. non-infected WT plants, as well as of infected *bzip28 bzip60* mutant vs. non-infected *bzip28 bzip60* mutant plants was performed, as reported in the main text and Figure 7.

Pathogen infection triggers extensive reprogramming of host cells, to fuel the synthesis of inducible components of the plant immunity machinery. To shed light on possible determinants of the reduced defense response of the UPR mutant, a comparative TMT label-based proteomic analysis of WT and *bzip28 bzip60* mutant samples in control conditions and 24 h after fungal infection was undertaken. This analysis allowed the identification and relative quantitation of 1745 *A. thaliana* proteins. When an abundance fold change threshold ≤0.66 or ≥1.50 (*p* ≤ 0.05) was considered for an independent pairwise comparison of leaves of infected WT vs. non-infected WT (WTinf/WT), as well as of infected *bzip28 bzip60* mutant vs. non-infected *bzip28 bzip60* mutant (*bzip28 bzip60* inf/*bzip28 bzip60*), 69 differentially represented proteins (DRPs) were identified. Quantitative proteomic results for DRPs are shown in a heat-map format in Figure 7. This figure also illustrates that 56 DRPs (39 over-represented and 17 under-represented) were detected in the WTinf/WT comparison, while 31 DRPs (22 over-represented and 9 under-represented) in the *bzip28 bzip60* inf/*bzip28 bzip60* one. Unique and shared DRPs are reported in a dedicated Venn diagram (Figure 8a). No significant differences in the protein repertoire were detected between *bzip28 bzip60* mutant and WT samples in the absence of *D. gigantea* infection (data not shown). Protein identification and abundance details are reported in Table S3.

Hierarchical clustering of abundance ratios and distribution of DRPs among different samples showed a general coherent quantitative trend in the two pairwise comparisons. However, the most significant changes in DRPs occurred in the WTinf/WT one. In addition to the higher number of unique DRPs in the WTinf/WT as compared to the *bzip28 bzip60* inf/*bzip28 bzip60* counterpart (38 vs. 13) (Figure 8a), common DRPs (18 in number) always showed greatest quantitative differences in WT plants, with only four exceptions. In proteomic terms, these findings demonstrate that the WT plants were more prone to change their protein repertoire to face fungal infection than the *bzip28 bzip60* ones.

The results of functional analysis of DRPs are shown in Figure 8b. They highlight significant molecular processes and metabolic pathways affected by *D. gigantea* infection, including in representation order: (i) proteins involved in response to external biotic/abiotic stresses; (ii) proteins related to reactions and protein modifications facing oxidative insult; (iii) molecules involved in protein folding and degradation; (iv) enzymes associated with anabolism/catabolism of amino acids; (v) enzymes involved in biosynthesis/transformation of secondary metabolites and phytohormones; (vi) molecules involved in biosynthesis of proteins; (vii) proteins involved in photosynthesis; and (viii) proteins with unknown function. Functional enrichment of DRPs for biological process and molecular function (GO) established the participation of most DRPs in the response to a spectrum of chemical stimuli, as well as in binding to ions/small molecules, redox reactions and glutathione metabolism, respectively (Tables S4 and S5).

Among the DRPs showing the highest over-representation after fungal challenge, three are worth mentioning, the gluthatione S-transferases (AT4G02520, AT1G02930 and AT1G02920), which are multifunctional enzymes inducible by different stress sources, including pathogens. These proteins act in the attenuation of oxidative stress and detoxification of toxic substances. Some GST isoforms have a peroxidase activity and function in the detoxification of membrane lipid hydroperoxides that accumulate during pathogen infection. Interestingly, it was recently reported that SA stimulates the expression of different GSTs [60]; our parallel measurements on relative GSTs and SA levels upon *D. gigantea* infection confirmed this observation. In the context of DRPs ensuring redox homeostasis can also be viewed the significant over-representation of three peroxidases (AT3G49120, AT4G08770 and AT4G37520) involved in H_2_O_2_ detoxification, toxic substance oxidation, lignin biosynthesis and auxin catabolism, which are induced by environmental stresses and pathogen infection [61]. Some of these proteins are cell wall peroxidases associated with cell wall stiffening upon pathogen infection. In the context of DRPs involved in stress response showing a significant over-representation upon *D. gigantea* challenge, the following species are also worth mentioning: (i) kunitz trypsin inhibitor 1 (AT1G73260), which was already reported being involved in modulating plant-pathogen interaction and PCD, and whose levels increase after H_2_O_2_ and SA stimulation [62]; (ii) endo-1,3-beta glucosidase (AT4G16260), which belongs to a class of enzymes participating in defense response against pathogens [63]; (iii) invertase/pectin methylesterase inhibitor (AT2G45220) which regulates pectin cell wall degradation and was reported to enhance resistance to infection by the fungus *Verticillium dahliae* [64]; (iv) the product of a senescence-associated gene (AT2G29350), whose transcription was reported to increase in plants treated with the intercellular fluid of *Arabidopsis* leaves infected by *B. cinerea* [65]; (v) a berberine-bridge enzyme-like species (AT1G26410) belonging to a family of proteins that oxidize oligogalacturonides produced during fungal infection, as a defense response aimed to limit available carbon sources to the pathogen [66]); and (vi) a lectin-like protein (AT3G15356) that shares structural homology with *Arabidopsis* COI1, a F-Box LRR motif-containing protein required for JA signaling and modulation of plant-pathogen interaction [67,68].

When STRING software was used to predict at medium confidence (0.4) an association map between *A. thaliana* DRPs related to *D. gigantea* infection, a main network connecting 44 components was observed, plus a single binary and quinary molecular complex (Figure S3 and Table S6). The predominant network involved about 64% of ascertained DRPs, suggesting the possible, coordinated regulation of various molecular processes and metabolic pathways following fungal challenge.

Overall, proteomic analysis demonstrated that *D. gigantea* infection elicited a similar response in both WT and *bzip28 bzip60* mutant plants, stimulating the production of a number of proteins related to defense and redox homeostasis and repressing the generation of molecules involved in protein synthesis and photosynthesis. Nevertheless, the response was qualitatively and quantitatively higher in WT plants, in accordance with their higher resistance to the pathogen.

### 3.9. D. gigantea Impaired the Downregulation of Defense-Related MicroRNA in bzip28 bzip60 Mutant Plants

**Figure 9:**
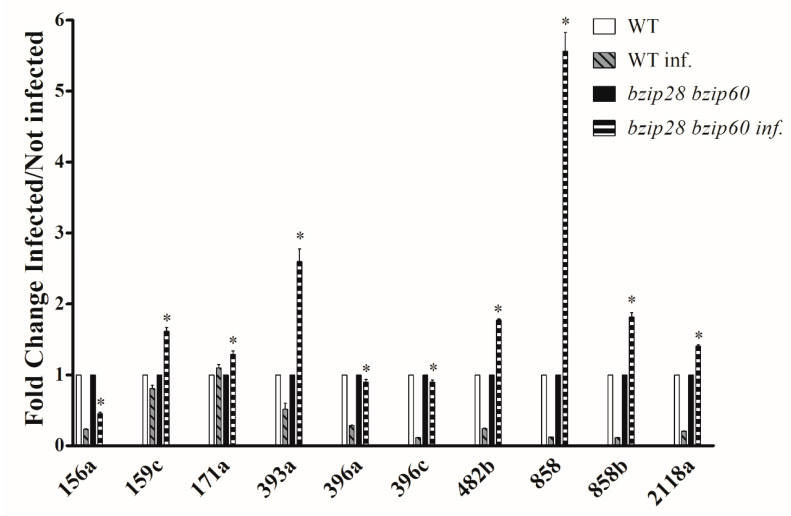
miRNA expression in leaves from WT and *bzip28 bzip68* mutant *Arabidopsis* plants infected by *D*. *gigantea* for 24 h. Accumulation of 156a, 159c, 171a, 393a, 396a, 396c, 482b, 858, 858b and 2118a miRNAs was determined by qRT-PCR in leaves from WT and *bzip28 bzip60* mutant *Arabidopsis* plants infected with a suspension of *D*. *gigantea* conidia (500,000/mL). After 24 h, leaves were collected and subjected to miRNA analysis as reported in Section 2.2. Values represent the mean values ± SD of independent measurements (*n* = 4). The results of the infected plants were reported as fold changes values with respect to the corresponding non-infected samples considered as unit (WT inf vs. WT; *bzip28 bzip60* inf vs. *bzip28 bzip60*). Statistical significance was attributed by one-way ANOVA test (* *p* < 0.05).

A growing body of evidence indicates that microRNAs are pivotal regulatory molecules of plant basal defense; they can be induced or repressed during pathogen infection to regulate the expression of defensive genes by mRNA cleavage or translational inhibition. To shed light on possible transcriptional regulatory mechanisms underlying the observed differences in the protein repertoire of WT vs. *bzip28 bzip60* mutant *Arabidopsis* plants infected by the fungus, the induction of a set of miRNAs already known as involved in plant defense was comparatively analyzed by qRT-PCR on leaves from WT and mutant plants infected for 24 h. The results show a quite different profile of miRNA expression between WT and mutant plants (Figure 9). The most remarkable quantitative differences concerned miR858 and miR858b, which were downregulated upon pathogen challenge in WT plants, whereas they were upregulated in mutant plants. MiR858 overexpression in *Arabidopsis* increased susceptibility to necrotrophic and hemibiotrophic fungi, whereas its downregulation by interference with target mimics (MIM858 plants) resulted in disease resistance [69].

The decrease of miR858 expression suggests that it may function as a negative regulator of the basal defense response. In fact, reduced activity of miR858 was previously shown to determine a strong activation of the defensive genes PDF1.2 and PR4, and produced an accumulation of phenylpropanoid compounds with antifungal activity in *Arabidopsis* leaves [69]. miR482 and miR2118 were upregulated in *bizip28 bzip60* mutant plants and downregulated in WT ones (Figure 9). These two miRNAs are components of a regulatory cascade targeting NBS-LRR proteins and affecting disease resistance in tomato [70]. The levels of miR396a and miR396c were reduced upon *D*. *gigantea* challenge in WT plants, whereas they were substantially unchanged in the *bizip28 bzip60* mutant plants. Interestingly, it has been shown that a reduction of miR396 activity with miRNA target mimics (MIM396 plants) increased the resistance of *Arabidopsis to* necrotrophic and hemibiotrophic fungi [71]. The expression of miR159c was slightly reduced in WT plants, while it was increased in *bizip28 bzip60* mutant plants. In *Arabidopsis*, impairment of miR159 activity was associated with an increased resistance to nematodes [72]. Finally, the expression of miR393 was increased upon *D. gigantea* infection in mutant plants, while it was decreased in WT plants. The upregulation of miR393 was previously shown to improve resistance to bacteria in *Arabidopsis*, a fact that was linked to the repression of auxin signaling [73]. Overall, these results demonstrate that UPR impairment during fungal infection severely affected the expression profile of several defense-related miRNAs; as correlated to the increased susceptibility of the UPR mutant *bzip28 bzip60* to the pathogen, this finding strongly suggests that the IRE1/bZIP60 pathway of UPR may affect basal defense response through a regulatory mechanism involving microRNAs.

## 4. Discussion

In this study, we investigated the involvement of UPR in the defense response of plants toward necrotrophic fungi. A comparative analysis of the elicitation of the defense response, and of UPR was performed in WT and in UPR-defective *bzip28bzip60* mutant *Arabidopsis* plants infected by *D. gigantea*. qRT-PCR results demonstrated that in WT plants the pathogen elicited a robust activation of the IRE1/bZIP60 pathway of UPR and that the functionality of this pathway was requested to fully activate the defense response. In fact, the *bzip28 bzip60* mutant, in which the UPR is constitutively hampered, was more susceptible to the fungal infection that provoked a reduced defense response and more severe symptoms and cellular damage than in WT plants. Interestingly, it has been reported that the expression of resistance of *N. attenuata* to the necrotrophic fungus *A. alternata* is dependent on the activation of the IRE1/bZIP60 pathway of UPR [27]. Hence, our data strongly suggest that elicitation of the UPR may be a common feature of the plant defense response to necrotrophic fungi.

Analysis of ER structure by confocal microscopy showed that the pathogen from early stages of infection brought about extensive ER network destruction and vacuolization, thereby demonstrating that the ER was a primary target of the fungal infection. Administration of exogenous OP-A demonstrated that ER damage was due to the release from penetrating hyphae of the phytotoxin, which destabilized the ER structure, hampering ER functionality and triggering UPR.

Proteomic analysis allowed estimating a reduced upregulation of a number of defense-related proteins or proteins involved in the maintenance of redox homeostasis in the *bzip28 bzip60* mutant after fungal challenge, a fact that is in line with increased symptoms, cellular damage and ROS over-production of the UPR-defective plants. Interestingly, most of the upregulated proteins were secreted proteins (peroxidase 37, 50 and 34, berberine-bridge enzyme-like 6, pectinesterase/pectinesterase inhibitor 17, and glucanendo-1,3-beta-glucosidase), ER resident polypeptides (glutathione-S-trasferase F2 and Kuniz-trypsin inhibitor) or components delivered to subcellular compartments (SAG 13, nitrilase, indole-3-glycerol phosphate synthase and tryptophane synthase). These findings underlie the role of ER and of the secretory pathway in the processing of components of the plant immunity system and provide a rationale for the impairment of the defense response in the *bzip28 bzip60* mutant.

Measurements of the concentration of the defensive hormone and UPR elicitor SA in leaves infected by *D. gigantea* showed that its levels were markedly increased in WT plants after 24 h, whereas much less in the *bzip28 bzip60* mutant. This result was confirmed by qRT-PCR analysis of the pathogen-inducible ICS1 gene of SA biosynthesis, showing that it was highly increased in WT plants but not in the *bzip28 bzip60* ones. This finding underlies the fact that SA, which is usually associated with defense against biotrophic fungi, can also be involved in the defense response against necrotrophic infections. Recent gene expression studies showed the involvement of both JA and SA signaling in the response to necrotrophic pathogens [58]. Exogenous SA was shown to increase the resistance of tomato leaves to *B. cynerea* [59], while tomato transgenic NahG plants, with reduced levels of SA, were more susceptible to the fungus [74]. Since it was reported that SA induced UPR genes when exogenously administered to *Arabidopsis* [55] or during pathogen infection [56], and that the induction involved activation of the IRE1/bZIP60 and bZIP28 pathways [55], our data showing that UPR impairment hampered SA biosynthesis let us hypothesize the occurrence of a reciprocal regulatory pathway, in which proper UPR is necessary to increase SA levels, a fact that in turn enhances UPR, according to a positive feedback mechanism.

The expression profile of a set of defense-related miRNAs resulted markedly different between WT and mutant plants. In the *bzip28 bzip60* mutant, the lack of downregulation of miRNAs appeared well correlated with a reduced synthesis of plant basal immunity proteins and increased susceptibility to the pathogen, thereby suggesting the occurrence of a regulatory link between ER functionality and modulation of basal defense. In fact, although in plants the contribution of miRNAs to UPR-dependent responses has been poorly investigated, in animals it has been shown that UPR sensors such as PERK, IRE1a, IRE1b and ATF6 can induce or suppress miRNAs, with a concomitant effect on cell fate or adaptation to stress [75,76].

## 5. Conclusions

Taking advantage of a comparative analysis of WT and UPR-defective *bzip28 bzip60* mutant *Arabidopsis* plants after *D. gigantea* infection, the present study demonstrated that OP-A secretion by the fungus targeted the ER structure, compromising its integrity and eliciting the IRE1/bZIP60 pathway of UPR that was necessary for a full activation of resistance to the necrotrophic pathogen. In fact, UPR impairment reduced the amount of extracellular defensive and oxidative-stress responsive proteins, hampered SA synthesis, and impaired defense-related microRNAs downregulation. These two latter findings underlie the occurrence of regulatory circuits between UPR and plant basal immunity that deserve further investigation.

## Data Availability

Proteomic data were deposited to the ProteomeXchange consortium via the PRIDE partner repository with the dataset identifier PXD018099. Other data will be made available by the authors upon request.

## Supplementary Materials

The following are available online at https://www.mdpi.com/2218-273X/11/2/240/s1, Figure S1: qRT-PCR analysis of UPR genes induction upon OP-A treatment of WT Arabidopsis leaves, Figure S2: HPLC detection of OP-A, Figure S3: STRING analysis of differential proteins, Table S1: list of primers of UPR genes, Table S2: list of primers of microRNAs, Table S3: identification parameters of differential proteins, Table S4: biological functions of Top 10 differential proteins, Table S5: molecular function of Top 10 differential proteins, Table S6: nodes identified in STRING analysis of differential proteins.
