# Supplementary material for: Arabidopsis Defense against the Pathogenic Fungus Drechslera gigantea Is Dependent on the Integrity of the Unfolded Protein Response
Source: Biomolecules. 2021 Feb 8;11(2):240. doi: 10.3390/biom11020240 (PMC7915340; doi:10.3390/biom11020240)
Supplement: Supplementary file 1 [file biomolecules-11-00240-s001.pdf]

Supplementary data

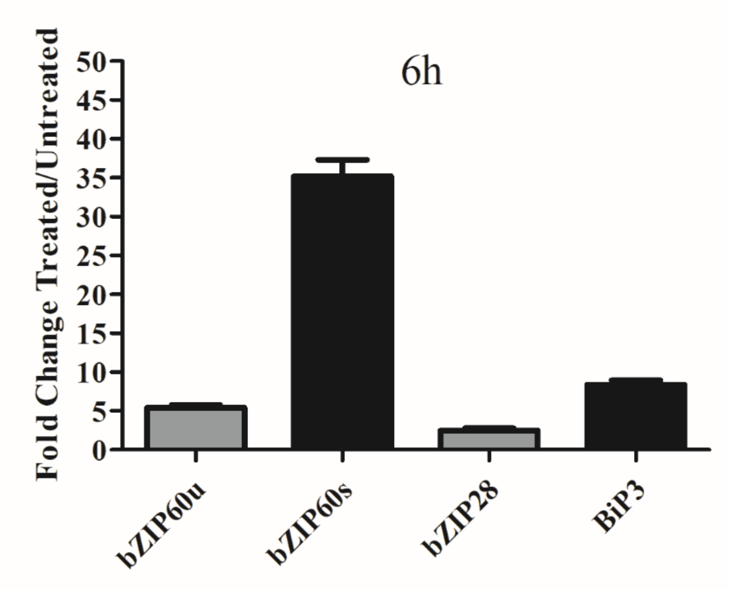

Supplementary Figure S1. UPR genes transcription in leaves from WT *Arabidopsis* plants incubated with OP-A Analysis of relative expression of, bZIP60u, bZIP60s, bZIP28 and BiP3 genes following incubation with 20 μM OP-A for 6 h. mRNA levels were quantified by qRT-PCR using ACT8 as housekeeping gene. Results from OP-A treated plants were reported as fold changes with respect to non-treated samples. Results represent the mean values ± SD of independent experiments (n=3). Samples were run in technical duplicates

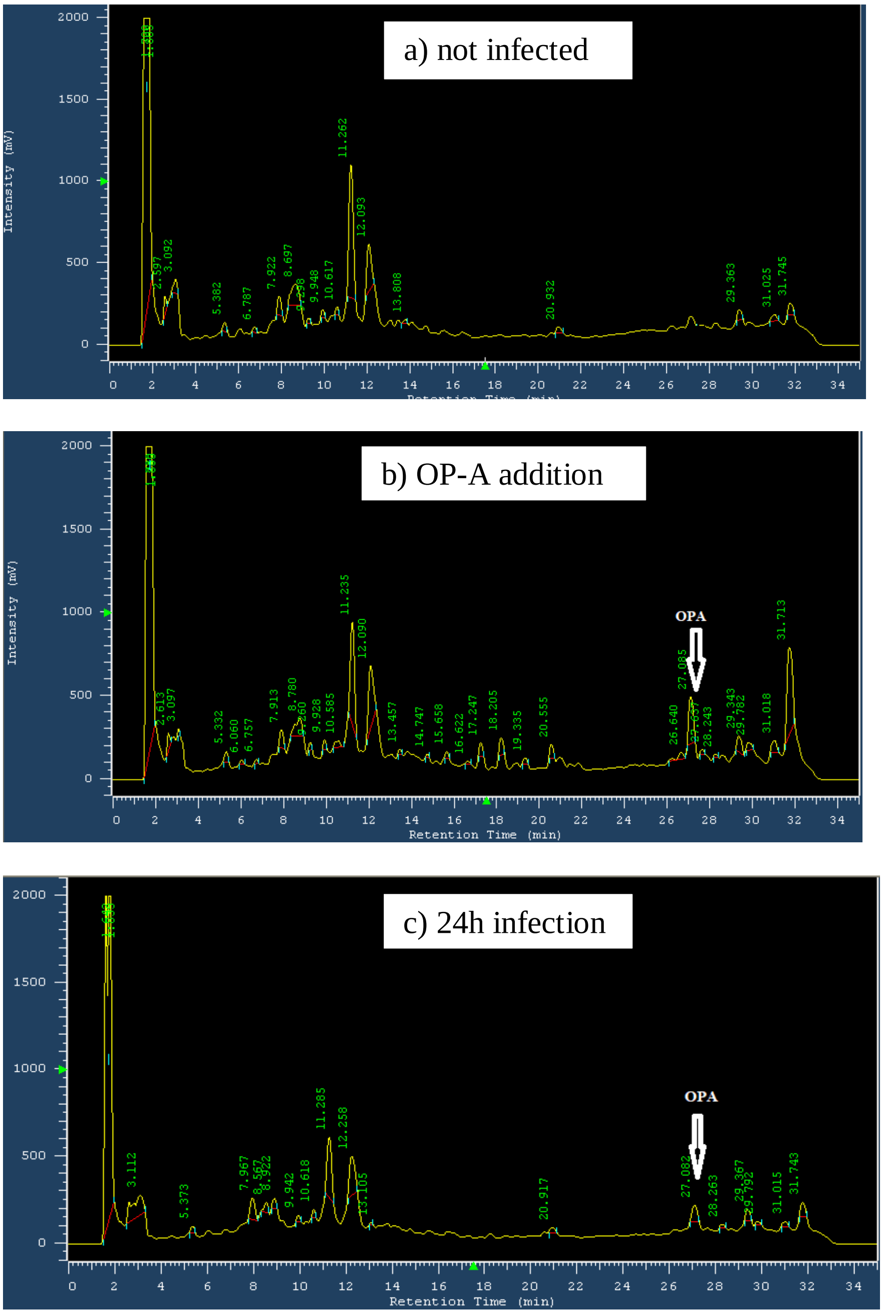

Supplementary Figure S2. HPLC determination of OP-A concentration in *Arabidopsis* leaves infected by *D. gigantea*. Methanolic extracts of WT *Arabidopsis* leaves, not infected, not infected and supplemented with exogenous OP-A or infected by a suspension of 500,000 conidia/ml of *D. gigantea* for 24 h were subjected to HPLC analysis as reported in 2.8 (a) chromatogram of extract from non infected leaves. (b) chromatogram of extract from non infected leaves supplemented with 10 µM OP-A (final concentration) (c) chromatogram of extract from infected leaves. Determined OP-A amounts were 16 µg in extract from non infected leaves containing 10 µM OP-A and 5.3 µg in extract from infected leaves.

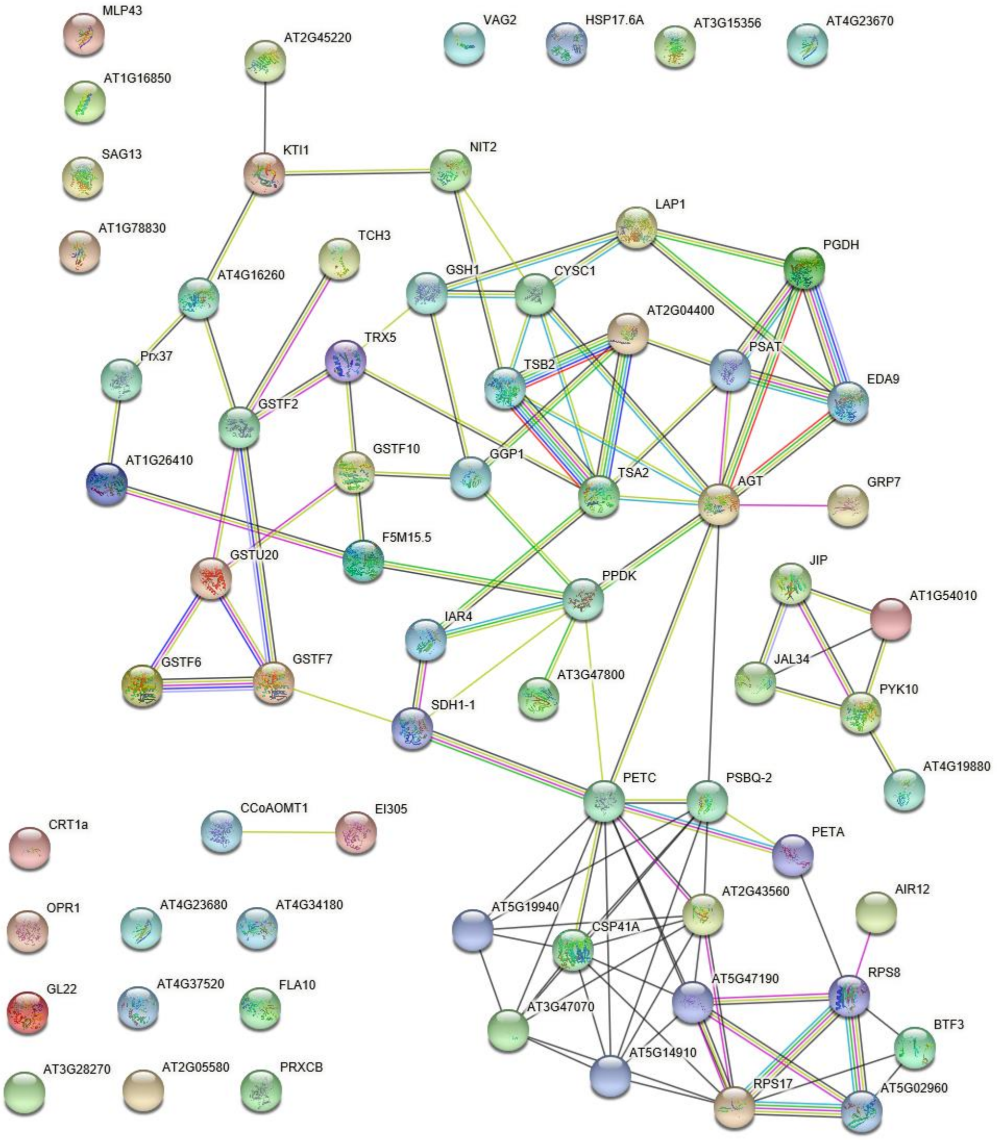

Supplementary Figure S3. STRING analysis of differentially represented proteins in leaves from WT and *bzip28 bzip60* mutant *Arabidopsis* plants infected or not by *D. gigantea* for 24 h. An independent pairwise comparison of leaves of infected WT *vs* non-infected WT plants as well as of infected *bzip28 bzip60* mutant *vs* non-infected *bzip28 bzip60* mutant plants was performed as reported in the main text**.** Abbreviations are reported in Supplementary Table S6.

Supplementary Table S1. Nucleotide primers used in qRT-PCR analysis for the quantitation of UPR gene expression.

Gene	Primer sense 5'-3'	Primer antisense 5'-3'
ACT8	TCAGCACTTTCCAGCAGATG	ATGCCTGGACCTGCTTCAT
BiP1	TCAGTCCTGAGGAGATTAGTGCT	TGCCTTTGAGCATCATTGAA
BiP3	CGAAACGTCTGATTGGAAGAA	GGCTTCCCATCTTTGTTCAC
bZIP60s	AAGCAGGAGTCTGCTGTTGG	TTTGTGTGGGACATATAAGGGAAT
bZIP60u	AGTCTGCTGTGCTCTTGTTGG	AGTCTGCTGTGCTCTTGTTGG
IRE1a	GCGCTACAGGCGTTACAAATA	TCGTCGAATCCTTCTGGAACT
IRE1b	AGTGGGGAAAAACCAGTTCC	AACCAAGTCTCGGAAACAGTG
bZIP28	CAGCAATGCTTCCGCATTCA	ATCCATTAGTGGCCCTGAG
bZIP17	TGAAGGTGTTGCAGGTCCC	ACGTTGGTAGCTGCAGGAAT
CNX1	AGCCTGAGGTTTCCTTTTGC	ACAGAACCGTTTGATCGTCAC
SAG12	GTGTCTACGCGGATGTGAAG	CAGCAAACTGATTTACCGCA
BI-1	CTCTTGTGGCGTCTGCCTTT	CGTTGTAAGAATACCGCCGATAT
PR1	CGAGAAGGCTAACTACAACTACG	ACACCTCACTTTGGCACATC
ICS1	CTCTCCAACTCCATTCACTA	TCTCCATCACAACCATTCA

Supplementary Table S2. Nucleotide primers used in qRT-PCR analysis for the quantitation of microRNA expression.

microRNA	Bait sequence 5'-3'
156a	UGACAGAAGAGAGUGAGCAC
159c	GAAUUCCUUCUCCUCU
171a	UGAUUGAGCCGCGCCAAUAUC
393a	UCCAAAGGGAUCGCAUUGAUC
396a	UUCCACAGCUUUCUUGAACGU
396c	UUCCACAGCUUUCUUGAACUU
482b	UCUUUCCUAUCCCUCCCAUUCC
858	UUCGUUGUCUGUUCGACCUGA
858b	UUCGUUGUCUGUUCGACCUU
2118a	CUACCGAUGCCACUAAGUCCCA

Supplementary Table S3. Relative quantitative levels, identification parameters and functional information of differentially represented proteins in leaves from WT and *bzip28 bzip60* mutant *Arabidopsis* plants infected or not by *D. gigantea* for 24 h. Results derive from an independent pairwise comparison of leaves of infected WT *vs* non-infected WT plants as well as of infected *bzip28 bzip60* mutant *vs* non-infected *bzip28 bzip60* mutant plants. In the Table Wt infected *vs* WT control, the identification and relative quantification details of the DRPs (56 in number) in leaves from WT *Arabidopsis* plants infected by *D. gigantea* for 24 h, with respect to non-infected counterparts are reported. In the Table *bzip28 bzip60* infected *vs* bzip28 bzip60 control, the identification and quantification details of the DRPs (31 in number) in leaves from *bzip28 bzip60* mutant *Arabidopsis* plants infected by *D. gigantea* for 24 h with respect to non-infected counterparts are reported. In the Table 69 DPRs Mercator, the functional assignment of all DRPs across MapMan BINs categories is reported. In the Table Blast 69 DRPs, the output of the Blast analysis of all DRPs against the *Viridiplantae* protein sequences from UniProtKB database is reported.

Supplementary Table S3 : Wt infected vs WT control

Accession number and protein description from TAIR10 database, number of identified peptides, peptide spectrum matches (PSMs), abundance ratio, abundance ratio p-value, MapMan Bin code and description of the functional categories assigned by Mercator analysis are provided. The accession numbers assigned to more than one functional category are colored in the Table

Accession	Description	# Peptides	# PSMs	Abundance Ratio: (129N,Wt infected) / (128N, WT Contr)	Abundance Ratio P-Value: (129N,Wt infected) / (128N, WT Contr)	BINCODE	NAME
AT4G16260.1	Glycosyl hydrolase superfamily protein	8	21	3.881	2.63964E-07	'50.3.2'	'Enzyme classification.EC_3 hydrolases.EC_3.2 glycosylase'
AT3G44300.1	nitrilase 2	9	41	2.986	7.12918E-07	'9.5.2.5'	'Secondary metabolism.glucosinolates.glucosinolate degradation.nitrilase'
AT4G02520.1	glutathione S-transferase PHI 2	13	141	4.193	7.16058E-06	'18.8.1.5'	'Protein modification.S-glutathionylation.glutathione S-transferase activities.class phi glutathione S-transferase'
AT2G29350.1	senescence-associated gene 13	6	15	3.132	1.25948E-05	'50.1.1'	'Enzyme classification.EC_1 oxidoreductases.EC_1.1 oxidoreductase acting on CH-OH group of donor'
AT1G02930.1	glutathione S-transferase 6	10	131	4.752	2.45324E-05	'18.8.1.5'	'Protein modification.S-glutathionylation.glutathione S-transferase activities.class phi glutathione S-transferase'
AT1G78830.1	Curculin-like (mannose-binding) lectin family protein	20	88	1.564	2.57069E-05	'35.1'	'not assigned.annotated'
AT3G61440.1	cysteine synthase C1	9	41	1.799	0.000115324	'50.2.5'	'Enzyme classification.EC_2 transferases.EC_2.5 transferase transferring alkyl or aryl group, other than methyl group'
AT1G45145.1	thioredoxin H-type 5	5	24	1.867	0.000174762	'18.7.2'	'Protein modification.S-nitrosylation.protein-S-nitrosothiol reductase (TRX5)'
AT1G45145.1	thioredoxin H-type 5	5	24	1.867	0.000174762	'10.6.1'	'Redox homeostasis.cytosol/mitochondrion/nucleus redox homeostasis.H-type thioredoxin'
AT3G15356.1	Legume lectin family protein	9	47	3.474	0.000201961	'18.4.1.19'	'Protein modification.phosphorylation.TKL protein kinase superfamily.protein kinase (L-lectin)'
AT1G20620.1	catalase 3	21	111	1.605	0.000308612	'10.2.1'	'Redox homeostasis.enzymatic reactive oxygen species scavengers.catalase'
AT3G49120.1	peroxidase CB	13	72	2.293	0.000462483	'35.1'	'not assigned.annotated'
AT1G02920.1	glutathione S-transferase 7	11	106	6.724	0.000779243	'18.8.1.5'	'Protein modification.S-glutathionylation.glutathione S-transferase activities.class phi glutathione S-transferase'
AT4G37520.1	Peroxidase superfamily protein	4	7	3.858	0.000940205	'35.1'	'not assigned.annotated'
AT2G04400.1	Aldolase-type TIM barrel family protein	9	22	2.808	0.001219598	'4.1.5.3.4'	'Amino acid metabolism.biosynthesis.shikimate family.tryptophan.indole-3-glycerol phosphate synthase'
AT4G23100.1	glutamate-cysteine ligase	6	14	1.862	0.001398655	'10.3.3.1.1'	'Redox homeostasis.low-molecular-weight scavengers.glutathione metabolism.glutathione biosynthesis.gamma-glutamyl:cysteine ligase'
AT4G34180.1	Cyclase family protein	7	27	1.789	0.001591519	'35.1'	'not assigned.annotated'
AT1G17745.2	D-3-phosphoglycerate dehydrogenase	10	23	1.517	0.001860347	'4.1.4.1.1'	'Amino acid metabolism.biosynthesis.serine family.non-photorespiratory serine.phosphoglycerate dehydrogenase'
AT1G56340.1	calreticulin 1a	7	15	1.765	0.00267575	'19.1.1.1.1.2'	Protein homeostasis.protein quality control.ER Quality Control (ERQC) machinery.calnexin/calreticulin chaperone system
AT1G24180.1	Thiamin diphosphate-binding fold (THDP-binding) superfamily protein	3	6	1.659	0.002982112	'2.2.1.1.1'	'Cellular respiration.pyruvate oxidation.mitochondrial pyruvate dehydrogenase complex.component E1 heterodimer.subunit alpha'
AT4G27070.1	tryptophan synthase beta-subunit 2	9	26	1.855	0.003203329	'4.1.5.3.5.2'	'Amino acid metabolism.biosynthesis.shikimate family.tryptophan.tryptophan synthase heterodimer.subunit beta'
AT4G08770.1	Peroxidase superfamily protein	5	15	11.258	0.00322427	'35.1'	'not assigned.annotated'
AT4G30530.1	Class I glutamine amidotransferase-like superfamily protein	7	22	1.746	0.004008829	'19.4.1.5'	'Protein homeostasis.proteolysis.cysteine-type peptidase activities.C26-class gamma-glutamyl peptidase'
AT4G30530.1	Class I glutamine amidotransferase-like superfamily protein	7	22	1.746	0.004008829	'9.5.1.7'	'Secondary metabolism.glucosinolates.glucosinolate biosynthesis.gamma-glutamyl peptidase'
AT5G66760.1	succinate dehydrogenase 1-1	2	4	1.541	0.004164182	'2.3.6.1.1'	'Cellular respiration.tricarboxylic acid cycle.succinate dehydrogenase complex.components.flavoprotein component SDH1'
AT1G73260.1	kunitz trypsin inhibitor 1	5	12	5.103	0.005160652	'35.1'	'not assigned.annotated'
AT3G47800.1	Galactose mutarotase-like superfamily protein	6	23	1.623	0.007427089	'35.2'	'not assigned.not annotated'
AT4G34200.1	D-3-phosphoglycerate dehydrogenase	16	50	1.993	0.007952686	'4.1.4.1.1'	'Amino acid metabolism.biosynthesis.serine family.non-photorespiratory serine.phosphoglycerate dehydrogenase'
AT4G15530.5	pyruvate orthophosphate dikinase	12	20	1.597	0.009248521	'3.10.2.1'	'Carbohydrate metabolism.gluconeogenesis.pyruvate orthophosphate dikinase activity.pyruvate orthophosphate dikinase'
AT3G54640.1	tryptophan synthase alpha chain	7	22	2.135	0.013710121	'4.1.5.3.5.1'	'Amino acid metabolism.biosynthesis.shikimate family.tryptophan.tryptophan synthase heterodimer.subunit alpha'
AT4G35630.1	phosphoserine aminotransferase	11	26	1.899	0.015727243	'4.1.4.1.2'	'Amino acid metabolism.biosynthesis.serine family.non-photorespiratory serine.phosphoserine aminotransferase'
AT4G34050.1	S-adenosyl-L-methionine-dependent methyltransferases superfamily protein	8	28	1.694	0.017017136	'21.6.1.4'	'Cell wall organisation.lignin.monolignol biosynthesis.caffeoyl-CoA 3-O-methyltransferase (CCoA-OMT)'
AT2G45220.1	Plant invertase/pectin methylesterase inhibitor superfamily	5	6	2.357	0.018724768	'21.3.1.2.1'	'Cell wall organisation.pectin.homogalacturonan.modification and degradation.pectin methylesterase'
AT4G19880.2	Glutathione S-transferase family protein	2	3	1.654	0.019947875	'35.2'	'not assigned.not annotated'
AT4G23710.1	vacuolar ATP synthase subunit G2	2	5	1.656	0.03176159	'24.1.1.2.7'	'Solute transport.primary active transport.V-type ATPase complex.peripheral V1 subcomplex.subunit G'
AT1G26410.1	FAD-binding Berberine family protein	4	8	4.497	0.031956967	'50.1.1'	'Enzyme classification.EC_1 oxidoreductases.EC_1.1 oxidoreductase acting on CH-OH group of donor'
AT1G76680.2	12-oxophytodienoate reductase 1	6	13	1.719	0.03196432	'50.1.3'	'Enzyme classification.EC_1 oxidoreductases.EC_1.3 oxidoreductase acting on CH-CH group of donor'
AT2G41100.1	Calcium-binding EF hand family protein	3	7	2.703	0.033744983	'35.1'	'not assigned.annotated'
AT1G02335.1	germin-like protein subfamily 2 member 2 precursor	4	18	1.585	0.042134201	'35.1'	'not assigned.annotated'
AT2G24200.1	Cytosol aminopeptidase family protein	20	61	1.624	0.043140551	'19.4.5.6.3'	'Protein homeostasis.proteolysis.metallopeptidase activities.aminopeptidase activities.M17-class leucyl aminopeptidase (LAP)'
AT1G62380.1	ACC oxidase 2	5	11	1.884	0.044132127	'11.5.1.2'	'Phytohormone action.ethylene.biosynthesis.1-aminocyclopropane-1-carboxylate (ACC) oxidase'
AT5G14910.1	Heavy metal transport/detoxification superfamily protein	3	18	0.437	0.000109639	'17.7.2.3.3'	'Protein biosynthesis.organelle machinery.plastidial ribosome.plastidial ribosome-associated proteins.ribosome biogenesis factor (CRASS)'
AT4G05180.1	photosystem II subunit Q-2	8	104	0.571	0.000140031	'1.1.1.2.2.2.2'	Photosynthesis.photophosphorylation.photosystem II.PS-II complex.oxygen-evolving center (OEC) extrinsic proteins.
AT3G47070.1	LOCATED IN: thylakoid, chloroplast thylakoid membrane	6	44	0.603	0.000156849	'35.2'	'not assigned.not annotated'
AT5G47190.1	Ribosomal protein L19 family protein	2	4	0.473	0.000162222	'17.7.2.1.17'	'Protein biosynthesis.organelle machinery.plastidial ribosome.large ribosomal subunit proteome.component psRPL19'
AT1G70890.1	MLP-like protein 43	6	33	0.626	0.000195425	'35.1'	'not assigned.annotated'
AT2G43560.1	FKBP-like peptidyl-prolyl cis-trans isomerase family protein	6	12	0.596	0.000220333	'18.12.2'	'Protein modification.protein folding.protein folding catalyst (FKBP)'
AT3G60900.1	FASCICLIN-like arabinogalactan-protein 10	3	12	0.623	0.001921741	'21.4.1.1.3'	Cell wall organisation.cell wall proteins.hydroxyproline-rich glycoprotein activities.arabinogalactan-protein (AGP) activities
AT5G19940.1	Plastid-lipid associated protein PAP / fibrillin family protein	4	16	0.639	0.001948684	'35.1'	'not assigned.annotated'
AT3G63140.1	chloroplast stem-loop binding protein of 41 kDa	13	54	0.613	0.003499036	'16.12.1.5'	'RNA processing.organelle machinery.ribonuclease activities.endoribonuclease (CSP41)'
AT4G03280.1	photosynthetic electron transfer C	6	62	0.526	0.004247359	'1.1.2.3'	'Photosynthesis.photophosphorylation.cytochrome b6/f complex.Rieske iron-sulfur component PetC'
AT4G23670.1	Polyketide cyclase/dehydrase and lipid transport superfamily protein	10	115	0.556	0.005002655	'35.1'	'not assigned.annotated'
AT5G02960.1	Ribosomal protein S12/S23 family protein	4	17	0.541	0.006465377	'17.1.3.1.24'	'Protein biosynthesis.ribosome biogenesis.small ribosomal subunit (SSU).SSU proteome.component RPS23'
AT4G23680.1	Polyketide cyclase/dehydrase and lipid transport superfamily protein	3	15	0.432	0.007144162	'35.1'	'not assigned.annotated'
AT1G79850.1	ribosomal protein S17	5	18	0.554	0.012473174	'17.7.2.2.17'	'Protein biosynthesis.organelle machinery.plastidial ribosome.small ribosomal subunit proteome.component psRPS17'
AT5G12030.1	heat shock protein 17.6A	2	10	0.648	0.014266373	'19.1.8.2'	'Protein homeostasis.protein quality control.smallHsp holdase chaperone activities.class-C-II protein'
AT1G16880.1	uridylyltransferase-related	6	26	0.528	0.029221276	'35.1'	'not assigned.annotated'
AT1G54010.1	GDSL-like Lipase/Acylhydrolase superfamily protein	11	32	0.599	0.044815726	'35.1'	'not assigned.annotated'

Supplementary Table S3: bzip28 bzip60 infected vs bzip28 bzip60 control

Accession number and protein description from TAIR10 database, number of identified peptides, peptide spectrum matches (PSMs), abundance ratio, abundance ratio p-value, MapMan Bin code and description of the functional categories assigned by Mercator analysis are provided. The accession numbers assigned to more than one functional category are colored in the Table.

Accession	Description	# Peptides	# PSMs	Abundance Ratio: (127N, bzip28 bzip60 infected) / (126, bzip28 bzip60 Contr)	Abundance Ratio P-Value: (127N, bzip28 bzip60 infected) / (126, bzip28 bzip60 Contr)	BINCODE	NAME
AT1G78830.1	Curculin-like (mannose-binding) lectin family protein	20	88	1.774	3.33439E-06	'35.1'	'not assigned.annotated'
AT4G16260.1	Glycosyl hydrolase superfamily protein	8	21	2.297	9.91845E-06	'50.3.2'	'Enzyme classification.EC_3 hydrolases.EC_3.2 glycosylase'
AT2G30870.1	glutathione S-transferase PHI 10	8	61	1.759	1.0701E-05	'18.8.1.5'	'Protein modification.S-glutathionylation.glutathione S-transferase activities.class phi glutathione S-transferase'
AT2G30870.1	glutathione S-transferase PHI 10	8	61	1.759	1.0701E-05	'9.5.1.6.4'	'Secondary metabolism.glucosinolates.glucosinolate biosynthesis.benzenic and indolic core structure.glutathione S-transferase'
AT4G02520.1	glutathione S-transferase PHI 2	13	141	3.723	1.31353E-05	'18.8.1.5'	'Protein modification.S-glutathionylation.glutathione S-transferase activities.class phi glutathione S-transferase'
AT3G44300.1	nitrilase 2	9	41	1.815	7.20304E-05	'9.5.2.5'	'Secondary metabolism.glucosinolates.glucosinolate degradation.nitrilase'
AT1G02930.1	glutathione S-transferase 6	10	131	3.636	0.000144035	'18.8.1.5'	'Protein modification.S-glutathionylation.glutathione S-transferase activities.class phi glutathione S-transferase'
AT3G16460.1	Mannose-binding lectin superfamily protein	16	56	1.575	0.000324841	'35.1'	'not assigned.annotated'
AT3G61440.1	cysteine synthase C1	9	41	1.532	0.000788687	'50.2.5'	'Enzyme classification.EC_2 transferases.EC_2.5 transferase transferring alkyl or aryl group, other than methyl group'
AT3G16420.1	PYK10-binding protein 1	14	72	1.675	0.001138943	'35.1'	'not assigned.annotated'
AT3G15356.1	Legume lectin family protein	9	47	2.618	0.001331605	'18.4.1.19'	'Protein modification.phosphorylation.TKL protein kinase superfamily.protein kinase (L-lectin)'
AT1G45145.1	thioredoxin H-type 5	5	24	1.624	0.001390345	'18.7.2'	'Protein modification.S-nitrosylation.protein-S-nitrosothiol reductase (TRX5)'
AT1G45145.1	thioredoxin H-type 5	5	24	1.624	0.001390345	'10.6.1'	'Redox homeostasis.cytosol/mitochondrion/nucleus redox homeostasis.H-type thioredoxin'
AT3G09260.1	Glycosyl hydrolase superfamily protein	15	93	2.077	0.00169706	'50.3.2'	'Enzyme classification.EC_3 hydrolases.EC_3.2 glycosylase'
AT1G02920.1	glutathione S-transferase 7	11	106	5.004	0.002493273	'18.8.1.5'	'Protein modification.S-glutathionylation.glutathione S-transferase activities.class phi glutathione S-transferase'
AT4G37520.1	Peroxidase superfamily protein	4	7	2.828	0.005135174	'35.1'	'not assigned.annotated'
AT4G34180.1	Cyclase family protein	7	27	1.572	0.005734948	'35.1'	'not assigned.annotated'
AT3G47800.1	Galactose mutarotase-like superfamily protein	6	23	1.668	0.006278898	'35.2'	'not assigned.not annotated'
AT4G27070.1	tryptophan synthase beta-subunit 2	9	26	1.648	0.007119887	'4.1.5.3.5.2'	'Amino acid metabolism.biosynthesis.shikimate family.tryptophan.tryptophan synthase heterodimer.subunit beta'
AT3G49120.1	peroxidase CB	13	72	1.705	0.007222947	'35.1'	'not assigned.annotated'
AT3G07390.1	auxin-responsive family protein	4	12	1.59	0.018827285	'35.1'	'not assigned.annotated'
AT1G02335.1	germin-like protein subfamily 2 member 2 precursor	4	18	1.647	0.023711375	'35.1'	'not assigned.annotated'
AT1G73260.1	kunitz trypsin inhibitor 1	5	12	3.137	0.027854486	'35.1'	'not assigned.annotated'
AT1G54010.1	GDSL-like Lipase/Acylhydrolase superfamily protein	11	32	1.788	0.035888511	'35.1'	'not assigned.annotated'
AT2G05580.1	Glycine-rich protein family	4	10	0.275	1.87726E-06	'35.2'	'not assigned.not annotated'
AT5G47190.1	Ribosomal protein L19 family protein	2	4	0.398	3.12708E-05	'17.7.2.1.17'	'Protein biosynthesis.organelle machinery.plastidial ribosome.large ribosomal subunit proteome.component psRPL19'
AT1G78370.1	glutathione S-transferase TAU 20	9	36	0.55	0.000101134	'18.8.1.4'	'Protein modification.S-glutathionylation.glutathione S-transferase activities.class tau glutathione S-transferase'
AT1G78370.1	glutathione S-transferase TAU 20	9	36	0.55	0.000101134	'9.5.1.5.3'	'Secondary metabolism.glucosinolates.glucosinolate biosynthesis.aliphatic core structure.glutathione S-transferase'
ATCG00540.1	photosynthetic electron transfer A	12	61	0.625	0.001058429	'1.1.2.1'	'Photosynthesis.photophosphorylation.cytochrome b6/f complex.apocytochrome f component PetA'
AT2G13360.1	alanine:glyoxylate aminotransferase	10	33	0.591	0.01118837	'4.1.2.1.3'	'Amino acid metabolism.biosynthesis.aspartate family.asparagine.asparagine aminotransaminase'
AT2G13360.1	alanine:glyoxylate aminotransferase	10	33	0.591	0.01118837	'1.3.3.2'	'Photosynthesis.photorespiration.aminotransferase activities.serine-glyoxylate transaminase'
ATCG00770.1	ribosomal protein S8	2	5	0.586	0.015944421	'17.7.2.2.8'	'Protein biosynthesis.organelle machinery.plastidial ribosome.small ribosomal subunit proteome.component psRPS8'
AT1G17880.1	basic transcription factor 3	6	25	0.601	0.024992725	'19.1.2.1.2'	'Protein homeostasis.protein quality control.ribosome-associated chaperone activities.NAC chaperone heterodimer.subunit beta'
AT3G28270.2	Protein of unknown function (DUF677)	4	9	0.589	0.039401843	'20.6.1'	'Cytoskeleton organisation.cytoskeleton-plasma membrane-cell wall interface.integrin-like protein (AT14a)'
AT2G21660.1	cold, circadian rhythm, and rna binding 2	7	24	0.634	0.040645399	'16.7.2'	'RNA processing.RNA chaperone activities.RNA chaperone (RZ1|GR-RBP)'

Supplementary Table S3: 69 DPRs Mercator

Accession number from TAIR10 database, MapMan Bin code, name and description of the functional categories assigned by Mercator analysis are provided. This classification was further integrated with functional information from scientific literature (Bevan et al., 1998, and recent publications).

IDENTIFIER	BINCODE	NAME	DESCRIPTION
at2g13360.1	'4.1.2.1.3'	'Amino acid metabolism.biosynthesis.aspartate family.asparagine.asparagine aminotransaminase'	'asparagine aminotransaminase (original description: | Symbols:SGAT,AGT,AGT1 | ALANINE:GLYOXYLATE AMINOTRANSFERASE 1,alanine:glyoxylate aminotransferase,L-serine:glyoxylate aminotransferase | Chr2:5539417-5540902 REVERSE LENGTH=401)'
at4g34200.1	'4.1.4.1.1'	'Amino acid metabolism.biosynthesis.serine family.non-photorespiratory serine.phosphoglycerate dehydrogenase'	'phosphoglycerate dehydrogenase (original description: | Symbols:EDA9,PGDH1 | phosphoglycerate dehydrogenase 1,embryo sac development arrest 9 | Chr4:16374041-16376561 REVERSE LENGTH=603)'
at1g17745.2	'4.1.4.1.1'	'Amino acid metabolism.biosynthesis.serine family.non-photorespiratory serine.phosphoglycerate dehydrogenase'	'phosphoglycerate dehydrogenase (original description: | Symbols:PGDH2,PGDH | phosphoglycerate dehydrogenase 2,3-phosphoglycerate dehydrogenase | Chr1:6101157-6104979 FORWARD LENGTH=651)'
at4g35630.1	'4.1.4.1.2'	'Amino acid metabolism.biosynthesis.serine family.non-photorespiratory serine.phosphoserine aminotransferase'	'phosphoserine aminotransferase (original description: | Symbols:PSAT1 | phosphoserine aminotransferase 1 | Chr4:16904205-16905497 FORWARD LENGTH=430)'
at2g04400.1	'4.1.5.3.4'	'Amino acid metabolism.biosynthesis.shikimate family.tryptophan.indole-3-glycerol phosphate synthase'	'indole-3-glycerol phosphate synthase (original description: | Symbols:no symbol available | no full name available | Chr2:1531208-1533578 FORWARD LENGTH=402)'
at3g54640.1	'4.1.5.3.5.1'	'Amino acid metabolism.biosynthesis.shikimate family.tryptophan.tryptophan synthase heterodimer.subunit alpha'	'subunit alpha of tryptophan synthase complex (original description: | Symbols:TRP3,TSA1 | TRYPTOPHAN-REQUIRING 3,tryptophan synthase alpha chain | Chr3:20223331-20225303 REVERSE LENGTH=312)'
at4g27070.1	'4.1.5.3.5.2'	'Amino acid metabolism.biosynthesis.shikimate family.tryptophan.tryptophan synthase heterodimer.subunit beta'	'subunit beta of tryptophan synthase complex (original description: | Symbols:TSB2 | tryptophan synthase beta-subunit 2 | Chr4:13586564-13588619 FORWARD LENGTH=475)'
at4g15530.5	'3.10.2.1'	'Carbohydrate metabolism.gluconeogenesis.pyruvate orthophosphate dikinase activity.pyruvate orthophosphate dikinase'	'pyruvate orthophosphate dikinase (original description: | Symbols:PPDK | pyruvate orthophosphate dikinase | Chr4:8864828-8870748 REVERSE LENGTH=963)'
at3g60900.1	'21.4.1.1.3'	'Cell wall organisation.cell wall proteins.hydroxyproline-rich glycoprotein activities.arabinogalactan-protein (AGP) activities.fasciclin-type AGP (FLA)'	'fasciclin-type arabinogalactan protein (original description: | Symbols:FLA10 | FASCICLIN-like arabinogalactan-protein 10 | Chr3:22499573-22500841 REVERSE LENGTH=422)'
at4g34050.1	'21.6.1.4'	'Cell wall organisation.lignin.monolignol biosynthesis.caffeoyl-CoA 3-O-methyltransferase (CCoA-OMT)'	'caffeoyl-CoA 3-O-methyltransferase (CCoA-OMT) (original description: | Symbols:CCoAOMT1 | caffeoyl coenzyme A O-methyltransferase 1 | Chr4:16310844-16311973 FORWARD LENGTH=259)'

at2g45220.1	'21.3.1.2.1'	'Cell wall organisation.pectin.homogalacturonan.modification and degradation.pectin methylesterase'	'pectin methylesterase (original description: | Symbols:AtPME17,PME17 | pectin methylesterase 17 | Chr2:18644281-18646394 REVERSE LENGTH=511)'
at1g24180.1	'2.2.1.1.1'	'Cellular respiration.pyruvate oxidation.mitochondrial pyruvate dehydrogenase complex.component E1 heterodimer.subunit alpha'	'subunit alpha of pyruvate dehydrogenase E1 component subcomplex (original description: | Symbols:IAR4 | IAA-CONJUGATE-RESISTANT 4 | Chr1:8560777-8563382 REVERSE LENGTH=393)'
at5g66760.1	'2.3.6.1.1'	'Cellular respiration.tricarboxylic acid cycle.succinate dehydrogenase complex.components.flavoprotein component SDH1'	'flavoprotein component SDH1 of succinate dehydrogenase complex (original description: | Symbols:SDH1-1 | succinate dehydrogenase 1-1 | Chr5:26653776-26657224 FORWARD LENGTH=634)'
at3g28270.2	'20.6.1'	'Cytoskeleton organisation.cytoskeleton-plasma membrane-cell wall interface.integrin-like protein (AT14a)'	'integrin-like protein (AT14a) (original description: | Symbols:AFL1 | At14a-Like1 | Chr3:10538725-10539849 FORWARD LENGTH=374)'
at2g29350.1	'50.1.1'	'Enzyme classification.EC_1 oxidoreductases.EC_1.1 oxidoreductase acting on CH-OH group of donor'	'Senescence-associated protein 13 OS=Arabidopsis thaliana (sp|q9zw18|sag13_arath : 529.0) & Enzyme classification.EC_1 oxidoreductases.EC_1.1 oxidoreductase acting on CH-OH group of donor(50.1.1 : 411.9) (original description: | Symbols:SAG13 | senescence
at1g26410.1	'50.1.1'	'Enzyme classification.EC_1 oxidoreductases.EC_1.1 oxidoreductase acting on CH-OH group of donor'	'Berberine bridge enzyme-like 6 OS=Arabidopsis thaliana (sp|q9fzc7|fox4_arath : 1047.0) & Enzyme classification.EC_1 oxidoreductases.EC_1.1 oxidoreductase acting on CH-OH group of donor(50.1.1 : 446.3) (original description: | Symbols:AtBBE6 | | Chr1:91
at1g76680.2	'50.1.3'	'Enzyme classification.EC_1 oxidoreductases.EC_1.3 oxidoreductase acting on CH-CH group of donor'	'12-oxophytodienoate reductase 1 OS=Arabidopsis thaliana (sp|q8lah7|opr1_arath : 771.0) & Enzyme classification.EC_1 oxidoreductases.EC_1.3 oxidoreductase acting on CH-CH group of donor(50.1.3 : 687.0) (original description: | Symbols:OPR1,ATOPR1 | 12-oxo
at3g61440.1	'50.2.5'	'Enzyme classification.EC_2 transferases.EC_2.5 transferase transferring alkyl or aryl group, other than methyl group'	'Bifunctional L-3-cyanoalanine synthase/cysteine synthase C1, mitochondrial OS=Arabidopsis thaliana (sp|q9s757|cysc1_arath : 698.0) & Enzyme classification.EC_2 transferases.EC_2.5 transferase transferring alkyl or aryl group, other than methyl group(50.2
at4g16260.1	'50.3.2'	'Enzyme classification.EC_3 hydrolases.EC_3.2 glycosylase'	'Probable glucan endo-1,3-beta-glucosidase At4g16260 OS=Arabidopsis thaliana (sp|q8vzj2|bgnem_arath : 631.0) & Enzyme classification.EC_3 hydrolases.EC_3.2 glycosylase(50.3.2 : 446.7) (original description: | Symbols:no symbol available | no full name ava
at3g09260.1	'50.3.2'	'Enzyme classification.EC_3 hydrolases.EC_3.2 glycosylase'	'Beta-glucosidase 23 OS=Arabidopsis thaliana (sp|q9sr37|bgl23_arath : 1042.0) & Enzyme classification.EC_3 hydrolases.EC_3.2 glycosylase(50.3.2 : 395.9) (original description: | Symbols:LEB,BGLU23,PYK10,PSR3.1 | LONG ER BODY | Chr3:2840657-2843730 REVERS
at5g19940.1	'35.1'	'not assigned.annotated'	' (original description: | Symbols:no symbol available | no full name available | Chr5:6739693-6740661 FORWARD LENGTH=239) & Probable plastid-lipid-associated protein 8, chloroplastic OS=Arabidopsis thaliana (sp|q941d3|pap8_arath : 386.0)'
at4g37520.1	'35.1'	'not assigned.annotated'	' (original description: | Symbols:no symbol available | no full name available | Chr4:17631704-17633060 FORWARD LENGTH=329) & Peroxidase 50 OS=Arabidopsis thaliana (sp|q43731|per50_arath : 611.0)'

at4g34180.1	'35.1'	'not assigned.annotated'	' (original description: | Symbols:CYCLASE1 | CYCLASE1 | Chr4:16370060-16371383 REVERSE LENGTH=255) & no description available(sp|q93v74|cyl1_arath : 476.0)'
at4g23680.1	'35.1'	'not assigned.annotated'	' (original description: | Symbols:no symbol available | no full name available | Chr4:12336416-12337417 REVERSE LENGTH=151) & MLP-like protein 328 OS=Arabidopsis thaliana (sp|q9zvf3|ml328_arath : 238.0)'
at4g23670.1	'35.1'	'not assigned.annotated'	' (original description: | Symbols:no symbol available | no full name available | Chr4:12332846-12333656 REVERSE LENGTH=151) & MLP-like protein 328 OS=Arabidopsis thaliana (sp|q9zvf3|ml328_arath : 223.0)'
at4g08770.1	'35.1'	'not assigned.annotated'	' (original description: | Symbols:Prx37 | peroxidase 37 | Chr4:5598259-5600262 REVERSE LENGTH=346) & Peroxidase 37 OS=Arabidopsis thaliana (sp|q9ldn9|per37_arath : 664.0)'
at3g49120.1	'35.1'	'not assigned.annotated'	' (original description: | Symbols:PERX34,ATPERX34,PRX34,ATPCB,PRXCB | ARABIDOPSIS THALIANA PEROXIDASE CB,PEROXIDASE 34,peroxidase CB | Chr3:18207819-18210041 FORWARD LENGTH=353) & Peroxidase 34 OS=Arabidopsis thaliana (sp|q9smu8|per34_arath : 689.0)'
at3g16460.1	'35.1'	'not assigned.annotated'	' (original description: | Symbols:JAL34 | jacalin-related lectin 34 | Chr3:5593029-5595522 FORWARD LENGTH=705) & Jacalin-related lectin 34 OS=Arabidopsis thaliana (sp|o04310|jal34_arath : 816.0)'
at3g16420.1	'35.1'	'not assigned.annotated'	' (original description: | Symbols:PBPI,JAL30,PBP1 | PYK10-binding protein 1,JACALIN-RELATED LECTIN 30 | Chr3:5579560-5580674 FORWARD LENGTH=298) & PYK10-binding protein 1 OS=Arabidopsis thaliana (sp|o04314|jal30_arath : 508.0)'
at3g07390.1	'35.1'	'not assigned.annotated'	' (original description: | Symbols:AIR12 | Auxin-Induced in Root cultures 12 | Chr3:2365452-2366273 FORWARD LENGTH=273) & Auxin-induced in root cultures protein 12 OS=Arabidopsis thaliana (sp|q94bt2|air12_arath : 372.0)'
at2g41100.1	'35.1'	'not assigned.annotated'	' (original description: | Symbols:TCH3,CML12,ATCAL4 | ARABIDOPSIS THALIANA CALMODULIN LIKE 4,calmodulin-like 12,TOUCH 3 | Chr2:17138131-17139406 FORWARD LENGTH=324) & Calmodulin-like protein 12 OS=Arabidopsis thaliana (sp|p25071|cml12_arath : 559.0)'
at1g78830.1	'35.1'	'not assigned.annotated'	' (original description: | Symbols:no symbol available | no full name available | Chr1:29637141-29638508 REVERSE LENGTH=455) & EP1-like glycoprotein 2 OS=Arabidopsis thaliana (sp|q9zva2|ep1l2_arath : 866.0)'
at1g73260.1	'35.1'	'not assigned.annotated'	' (original description: | Symbols:ATKTI1,KTI1 | kunitz trypsin inhibitor 1,ARABIDOPSIS THALIANA KUNITZ TRYPSIN INHIBITOR 1 | Chr1:27547410-27548057 REVERSE LENGTH=215) & Kunitz trypsin inhibitor 1 OS=Arabidopsis thaliana (sp|q8rxd5|kti1_arath : 413.0)'
at1g70890.1	'35.1'	'not assigned.annotated'	' (original description: | Symbols:MLP43 | MLP-like protein 43,major latex protein like 43 | Chr1:26725912-26726489 REVERSE LENGTH=158) & MLP-like protein 43 OS=Arabidopsis thaliana (sp|q9ssk5|mlp43_arath : 286.0)'

at1g54010.1	'35.1'	'not assigned.annotated'	' (original description: | Symbols:GLL23 | GDSL-like lipase 23 | Chr1:20158854-20160747 REVERSE LENGTH=386) & Inactive GDSL esterase/lipase-like protein 23 OS=Arabidopsis thaliana (sp|q8w4h8|gdl19_arath : 763.0)'
at1g16880.1	'35.1'	'not assigned.annotated'	' (original description: | Symbols:ACR11 | ACT domain repeats 11 | Chr1:5773796-5776125 FORWARD LENGTH=290) & ACT domain-containing protein ACR11 OS=Arabidopsis thaliana (sp|q9fz47|acr11_arath : 543.0)'
at1g02335.1	'35.1'	'not assigned.annotated'	' (original description: | Symbols:PDGLP2,GL22 | PLASMODESMAL GERMIN-LIKE PROTEIN 2,germin-like protein subfamily 2 member 2 precursor | Chr1:463979-464876 REVERSE LENGTH=219) & Germin-like protein subfamily 2 member 2 OS=Arabidopsis thaliana (sp|q9fz27
at4g19880.2	'35.2'	'not assigned.not annotated'	' no hits & (original description: | Symbols:no symbol available | no full name available | Chr4:10784691-10786376 REVERSE LENGTH=382)'
at3g47800.1	'35.2'	'not assigned.not annotated'	' no hits & (original description: | Symbols:no symbol available | no full name available | Chr3:17634971-17636998 FORWARD LENGTH=358)'
at3g47070.1	'35.2'	'not assigned.not annotated'	' no hits & (original description: | Symbols:no symbol available | no full name available | Chr3:17337205-17337507 REVERSE LENGTH=100)'
at2g05580.1	'35.2'	'not assigned.not annotated'	' no hits & (original description: | Symbols:no symbol available | no full name available | Chr2:2055578-2056563 FORWARD LENGTH=302)'
atcg00540.1	'1.1.2.1'	'Photosynthesis.photophosphorylation.cytochrome b6/f complex.apocytochrome f component PetA'	'apocytochrome f component PetA of cytochrome b6/f complex (original description: | Symbols:PETA | photosynthetic electron transfer A | ChrC:61657-62619 FORWARD LENGTH=320)'
at4g03280.1	'1.1.2.3'	'Photosynthesis.photophosphorylation.cytochrome b6/f complex.Rieske iron-sulfur component PetC'	'Rieske iron-sulfur component PetC of cytochrome b6/f complex (original description: | Symbols:PGR1,PETC | PROTON GRADIENT REGULATION 1,photosynthetic electron transfer C | Chr4:1440314-1441717 FORWARD LENGTH=229)'
at4g05180.1	'1.1.1.2.2.2.2'	'Photosynthesis.photophosphorylation.photosystem II.PS-II complex.oxygen-evolving center (OEC) extrinsic proteins.Viridiplantae-specific components.component OEC16/PsbQ'	'component PsbQ of PS-II oxygen-evolving center (original description: | Symbols:PSBQ-2,PSBQ,PSII-Q | photosystem II subunit Q-2,PHOTOSYSTEM II SUBUNIT Q | Chr4:2672093-2673170 REVERSE LENGTH=230)'
at2g13360.1	'1.3.3.2'	'Photosynthesis.photorespiration.aminotransferase activities.serine-glyoxylate transaminase'	'serine-glyoxylate transaminase (original description: | Symbols:SGAT,AGT,AGT1 | ALANINE:GLYOXYLATE AMINOTRANSFERASE 1,alanine:glyoxylate aminotransferase,L-serine:glyoxylate aminotransferase | Chr2:5539417-5540902 REVERSE LENGTH=401)'
at1g62380.1	'11.5.1.2'	'Phytohormone action.ethylene.biosynthesis.1-aminocyclopropane-1-carboxylate (ACC) oxidase'	'1-aminocyclopropane-1-carboxylate (ACC) oxidase (original description: | Symbols:ATACO2,ACO2 | ACC oxidase 2 | Chr1:23082340-23084068 FORWARD LENGTH=320)'

at5g47190.1	'17.7.2.1.17'	'Protein biosynthesis.organelle machinery.plastidial ribosome.large ribosomal subunit proteome.component psRPL19'	'component psRPL19 of large ribosomal subunit proteome (original description: | Symbols:no symbol available | no full name available | Chr5:19164432-19166064 REVERSE LENGTH=229)'
at5g14910.1	'17.7.2.3.3'	'Protein biosynthesis.organelle machinery.plastidial ribosome.plastidial ribosome-associated proteins.ribosome biogenesis factor (CRASS)'	'ribosome biogenesis factor (CRASS) (original description: | Symbols:no symbol available | no full name available | Chr5:4823815-4825196 FORWARD LENGTH=178)'
at1g79850.1	'17.7.2.2.17'	'Protein biosynthesis.organelle machinery.plastidial ribosome.small ribosomal subunit proteome.component psRPS17'	'component psRPS17 of small ribosomal subunit proteome (original description: | Symbols:RPS17,PDE347,CS17,PRPS17 | PIGMENT DEFECTIVE 347,PLASTID RIBOSOMAL SMALL SUBUNIT PROTEIN 17,ribosomal protein S17 | Chr1:30041473-30041922 REVERSE LENGTH=149)'
atcg00770.1	'17.7.2.2.8'	'Protein biosynthesis.organelle machinery.plastidial ribosome.small ribosomal subunit proteome.component psRPS8'	'component psRPS8 of small ribosomal subunit proteome (original description: | Symbols:RPS8 | ribosomal protein S8 | ChrC:80068-80472 REVERSE LENGTH=134)'
at5g02960.1	'17.1.3.1.24'	'Protein biosynthesis.ribosome biogenesis.small ribosomal subunit (SSU).SSU proteome.component RPS23'	'component RPS23 of SSU proteome (original description: | Symbols:no symbol available | no full name available | Chr5:693280-694396 REVERSE LENGTH=142)'
at1g56340.1	'19.1.1.1.1.2'	'Protein homeostasis.protein quality control.ER Quality Control (ERQC) machinery.calnexin/calreticulin chaperone system.CNX-CRT cycle.lectin chaperone (CRT)'	'lectin chaperone (CRT) (original description: | Symbols:AtCRT1a,CRT1,CRT1a | calreticulin 1a,calreticulin 1 | Chr1:21090059-21092630 REVERSE LENGTH=425)'
at1g17880.1	'19.1.2.1.2'	'Protein homeostasis.protein quality control.ribosome-associated chaperone activities.NAC chaperone heterodimer.subunit beta'	'subunit beta of NAC ribosome-associated chaperone complex (original description: | Symbols:ATBTF3,BTF3 | basic transcription factor 3 | Chr1:6152572-6153425 REVERSE LENGTH=165)'
at5g12030.1	'19.1.8.2'	'Protein homeostasis.protein quality control.smallHsp holdase chaperone activities.class-C-II protein'	'class-C-II small heat-shock-responsive protein (original description: | Symbols:AT-HSP17.6A,HSP17.6A,HSP17.6 | HEAT SHOCK PROTEIN 17.6,heat shock protein 17.6A | Chr5:3884214-3884684 REVERSE LENGTH=156)'
at4g30530.1	'19.4.1.5'	'Protein homeostasis.proteolysis.cysteine-type peptidase activities.C26-class gamma-glutamyl peptidase'	'gamma-glutamyl peptidase (original description: | Symbols:GGP1 | gamma-glutamyl peptidase 1 | Chr4:14920605-14922286 FORWARD LENGTH=250)'
at2g24200.1	'19.4.5.6.3'	'Protein homeostasis.proteolysis.metallopeptidase activities.aminopeptidase activities.M17-class leucyl aminopeptidase (LAP)'	'M17-class leucyl aminopeptidase (LAP) (original description: | Symbols:LAP1,atLAP1 | leucyl aminopeptidase 1 | Chr2:10287017-10289450 REVERSE LENGTH=520)'
at3g15356.1	'18.4.1.19'	'Protein modification.phosphorylation.TKL protein kinase superfamily.protein kinase (L-lectin)'	'protein kinase (L-lectin) (original description: | Symbols:no symbol available | no full name available | Chr3:5174603-5175418 REVERSE LENGTH=271)'
at2g43560.1	'18.12.2'	'Protein modification.protein folding.protein folding catalyst (FKBP)'	'protein folding catalyst (FKBP) (original description: | Symbols:no symbol available | no full name available | Chr2:18073995-18075385 REVERSE LENGTH=223)'

at4g02520.1	'18.8.1.5'	'Protein modification.S-glutathionylation.glutathione S-transferase activities.class phi glutathione S-transferase'	'class phi glutathione S-transferase (original description: | Symbols:ATGSTF2,GSTF2,ATPM24.1,GST2,ATPM24 | glutathione S-transferase PHI 2 | Chr4:1110673-1111531 REVERSE LENGTH=212)'
at2g30870.1	'18.8.1.5'	'Protein modification.S-glutathionylation.glutathione S-transferase activities.class phi glutathione S-transferase'	'class phi glutathione S-transferase (original description: | Symbols:GSTF10,ATGSTF10,ERD13,ATGSTF4 | glutathione S-transferase PHI 10,ARABIDOPSIS THALIANA GLUTATHIONE S-TRANSFERASE PHI 10,EARLY DEHYDRATION-INDUCED 13 | Chr2:13141490-13142392 FORWARD LEN
at1g02930.1	'18.8.1.5'	'Protein modification.S-glutathionylation.glutathione S-transferase activities.class phi glutathione S-transferase'	'class phi glutathione S-transferase (original description: | Symbols:ATGSTF3,ATGSTF6,GST1,ERD11,GSTF6,ATGST1 | EARLY RESPONSIVE TO DEHYDRATION 11,ARABIDOPSIS GLUTATHIONE S-TRANSFERASE 1,ARABIDOPSIS THALIANA GLUATIONE S-TRANSFERASE F3,glutathione S-transf
at1g02920.1	'18.8.1.5'	'Protein modification.S-glutathionylation.glutathione S-transferase activities.class phi glutathione S-transferase'	'class phi glutathione S-transferase (original description: | Symbols:ATGSTF8,GSTF7,ATGSTF7,ATGST11,GST11 | GLUTATHIONE S-TRANSFERASE 11,glutathione S-transferase 7,ARABIDOPSIS GLUTATHIONE S-TRANSFERASE 11 | Chr1:658886-659705 REVERSE LENGTH=209)'
at1g78370.1	'18.8.1.4'	'Protein modification.S-glutathionylation.glutathione S-transferase activities.class tau glutathione S-transferase'	'class tau glutathione S-transferase (original description: | Symbols:ATGSTU20,GSTU20 | glutathione S-transferase TAU 20 | Chr1:29484428-29485204 REVERSE LENGTH=217)'
at1g45145.1	'18.7.2'	'Protein modification.S-nitrosylation.protein-S-nitrosothiol reductase (TRX5)'	'protein-S-nitrosothiol reductase (TRX5) (original description: | Symbols:TRX-h5,ATTRX5,LIV1,ATH5,TRX5 | thioredoxin H-type 5,THIOREDOXIN H-TYPE 5,LOCUS OF INSENSITIVITY TO VICTORIN 1 | Chr1:17075264-17076256 REVERSE LENGTH=118)'
at1g45145.1	'10.6.1'	'Redox homeostasis.cytosol/mitochondrion/nucleus redox homeostasis.H-type thioredoxin'	'H-type thioredoxin (original description: | Symbols:TRX-h5,ATTRX5,LIV1,ATH5,TRX5 | thioredoxin H-type 5,THIOREDOXIN H-TYPE 5,LOCUS OF INSENSITIVITY TO VICTORIN 1 | Chr1:17075264-17076256 REVERSE LENGTH=118)'
at1g20620.1	'10.2.1'	'Redox homeostasis.enzymatic reactive oxygen species scavengers.catalase'	'catalase (original description: | Symbols:SEN2,ATCAT3,CAT3 | SENESCENCE 2,catalase 3 | Chr1:7143142-7146193 FORWARD LENGTH=492)'
at4g23100.1	'10.3.3.1.1'	'Redox homeostasis.low-molecular-weight scavengers.glutathione metabolism.glutathione biosynthesis.gamma-glutamyl:cysteine ligase'	'gamma-glutamyl:cysteine ligase (original description: | Symbols:CAD2,GSHA,RML1,AtGSH1,PAD2,ATECS1,GSH1 | PHYTOALEXIN DEFICIENT 2,glutamate-cysteine ligase,CADMIUM SENSITIVE 2,ROOT MERISTEMLESS 1 | Chr4:12103458-12106751 REVERSE LENGTH=522)'
at3g63140.1	'16.12.1.5'	'RNA processing.organelle machinery.ribonuclease activities.endoribonuclease (CSP41)'	'endoribonuclease (CSP41) (original description: | Symbols:CSP41A | chloroplast stem-loop binding protein of 41 kDa | Chr3:23327006-23328620 REVERSE LENGTH=406)'
at2g21660.1	'16.7.2'	'RNA processing.RNA chaperone activities.RNA chaperone (RZ1|GR-RBP)'	'RNA chaperone (RZ1|GR-RBP) (original description: | Symbols:GRP7,CCR2,GR-RBP7,ATGRP7,RBGA3 | RNA-binding glycine-rich protein A3,GLYCINE RICH PROTEIN 7,"cold, circadian rhythm, and rna binding 2",GLYCINE-RICH RNA-BINDING PROTEIN 7 | Chr2:9265477-9266316
at1g78370.1	'9.5.1.5.3'	'Secondary metabolism.glucosinolates.glucosinolate biosynthesis.aliphatic core structure.glutathione S-transferase'	'glutathione S-transferase (original description: | Symbols:ATGSTU20,GSTU20 | glutathione S-transferase TAU 20 | Chr1:29484428-29485204 REVERSE LENGTH=217)'

at2g30870.1	'9.5.1.6.4'	'Secondary metabolism.glucosinolates.glucosinolate biosynthesis.benzenic and indolic core structure.glutathione S-transferase'	'glutathione S-transferase (original description: | Symbols:GSTF10,ATGSTF10,ERD13,ATGSTF4 | glutathione S-transferase PHI 10,ARABIDOPSIS THALIANA GLUTATHIONE S-TRANSFERASE PHI 10,EARLY DEHYDRATION-INDUCED 13 | Chr2:13141490-13142392 FORWARD LENGTH=215)'
at4g30530.1	'9.5.1.7'	'Secondary metabolism.glucosinolates.glucosinolate biosynthesis.gamma-glutamyl peptidase'	'gamma-glutamyl peptidase (original description: | Symbols:GGP1 | gamma-glutamyl peptidase 1 | Chr4:14920605-14922286 FORWARD LENGTH=250)'
at3g44300.1	'9.5.2.5'	'Secondary metabolism.glucosinolates.glucosinolate degradation.nitrilase'	'nitrilase (original description: | Symbols:AtNIT2,NIT2 | nitrilase 2 | Chr3:15983351-15985172 FORWARD LENGTH=339)'
at4g23710.1	'24.1.1.2.7'	'Solute transport.primary active transport.V-type ATPase complex.peripheral V1 subcomplex.subunit G'	'subunit G of V-type ATPase peripheral V1 subcomplex (original description: | Symbols:VHA-G2,VAG2,VATG2 | VACUOLAR ATP SYNTHASE SUBUNIT G2,vacuolar ATP synthase subunit G2 | Chr4:12350577-12351354 FORWARD LENGTH=106)'

Supplementary Table S3: Blast 69 DRPs

Accession number from TAIR10 database, accession number, protein description and gene names from UniProtKB database, % identical matches, alignment length, number of mismatches, number of gap openings, start of alignment in query, end of alignment in query, start of alignment in subject, end of alignment in subject, expect value and bit score are provided.

TAIR 10 (query)	Uniprot		Uniprot Description	Uniprot gene names	% identity	alignment length	mismatches	gap	query start	query end	subject start	subject end	e-value	bit score
AT1G02335.1	sp|Q9FZ27|GL22_ARATH	reviewed	Germin-like protein subfamily 2 member 2	At1g02335 T6A9.29 T6A9.3	100	219	0	0	1	219	1	219	1.3E-158	447
AT1G02920.1	sp|Q9SRY5|GSTF7_ARATH	reviewed	Glutathione S-transferase F7 (EC 2.5.1.18) (AtGSTF8) (GST class-phi member 7) (Glutathione S-transferase 11)	GSTF7 GST11 GSTF8 At1g02920 F22D16.8	100	209	0	0	1	209	1	209	1.7E-150	426
AT1G02930.1	sp|P42760|GSTF6_ARATH	reviewed	Glutathione S-transferase F6 (AtGSTF6) (EC 2.5.1.18) (AtGSTF3) (GST class-phi member 6) (Glutathione S-transferase 1) (AtGST1) (Protein EARLY RESPONSE TO DEHYDRATION 11)	GSTF6 ERD11 GST1 GSTF3 At1g02930 F22D16.7	100	208	0	0	1	208	1	208	2.3E-150	425
AT1G16880.1	sp|Q9FZ47|ACR11_ARATH	reviewed	ACT domain-containing protein ACR11 (Protein ACT DOMAIN REPEATS 11)	ACR11 At1g16880 F6I1.12	100	290	0	0	1	290	1	290	0	583
AT1G17745.2	sp|O04130|SERA2_ARATH	reviewed	D-3-phosphoglycerate dehydrogenase 2, chloroplastic (PGDH) (EC 1.1.1.95)	PGDH2 3-PGDH PGDH At1g17745 F11A6.8	95.545	651	2	2	1	651	1	624	0	1244
AT1G17880.1	sp|Q9SMW7|BTF3_ARATH	reviewed	Basic transcription factor 3 (AtBTF3) (Nascent polypeptide-associated complex subunit beta)	BTF3 At1g17880 F2H15.11	100	165	0	0	1	165	1	165	7.9E-116	334
AT1G20620.1	sp|Q42547|CATA3_ARATH	reviewed	Catalase-3 (EC 1.11.1.6)	CAT3 At1g20620 F2D10.40 F5M15.5	100	492	0	0	1	492	1	492	0	1031
AT1G24180.1	sp|Q8H1Y0|ODPA2_ARATH	reviewed	Pyruvate dehydrogenase E1 component subunit alpha-2, mitochondrial (PDHE1-A) (EC 1.2.4.1) (Protein IAA-CONJUGATE-RESISTANT 4)	IAR4 At1g24180 F3I6.11	100	393	0	0	1	393	1	393	0	818
AT1G26410.1	sp|Q9FZC7|FOX4_ARATH	reviewed	Berberine bridge enzyme-like 6 (AtBBE-like 6) (EC 1.1.1.-) (Flavin-dependent oxidoreductase FOX4) (EC 1.-.-.-)	FOX4 At1g26410 T1K7.21	100	552	0	0	1	552	1	552	0	1139
AT1G45145.1	sp|Q39241|TRXH5_ARATH	reviewed	Thioredoxin H5 (AtTrxh5) (Protein LOCUS OF INSENSITIVITY TO VICTORIN 1) (Thioredoxin 5) (AtTRX5)	TRX5 LIV1 At1g45145 F27F5.21	100	118	0	0	1	118	1	118	5.95E-80	239
AT1G54010.1	sp|Q8W4H8|GDL19_ARATH	reviewed	Inactive GDSL esterase/lipase-like protein 23 (GDSL-like lipase 23) (Probable myrosinase-associated protein GLL23)	GLL23 At1g54010 F15I1.9	100	386	0	0	1	386	1	386	0	805
AT1G56340.1	sp|O04151|CALR1_ARATH	reviewed	Calreticulin-1	CRT1 At1g56340 F13N6.20 F14G9.5	100	425	0	0	1	425	1	425	0	853
AT1G62380.1	sp|Q41931|ACCO2_ARATH	reviewed	1-aminocyclopropane-1-carboxylate oxidase 2 (ACC oxidase 2) (AtACO2) (EC 1.14.17.4)	ACO2 EI305 At1g62380 F24O1.10	100	320	0	0	1	320	1	320	0	666
AT1G70890.1	sp|Q9SSK5|MLP43_ARATH	reviewed	MLP-like protein 43	MLP43 At1g70890 F15H11.12	100	158	0	0	1	158	1	158	1.3E-110	320

AT1G73260.1	sp|Q8RXD5|KTI1_ARATH	reviewed	Kunitz trypsin inhibitor 4 (AtKTI4) (Kunitz trypsin inhibitor 1) (AtKTI1) (Trypsin protease inhibitor)	KTI4 KTI1 TPI At1g73260 T18K17.7	100	215	0	0	1	215	1	215	1.3E-158	447
AT1G76680.2	tr|F4I403|F4I403_ARATH	unreviewed	12-oxophytodienoate reductase 1	OPR1 ATOPR1 At1g76680 F28O16.5 F28O16_5	100	397	0	0	1	397	1	397	0	829
AT1G78370.1	sp|Q8L7C9|GSTUK_ARATH	reviewed	Glutathione S-transferase U20 (AtGSTU20) (EC 2.5.1.18) (FIN219-interacting protein 1) (GST class-tau member 20)	GSTU20 FIP1 At1g78370 F3F9.11	100	217	0	0	1	217	1	217	1.6E-157	444
AT1G78830.1	sp|Q9ZVA2|EP1L2_ARATH	reviewed	EP1-like glycoprotein 2 (Curculin-like (Mannose-binding) lectin family protein)	At1g78830 F9K20.12	100	455	0	0	1	455	1	455	0	942
AT1G79850.1	sp|P16180|RR17_ARATH	reviewed	30S ribosomal protein S17, chloroplastic (CS17)	RPS17 At1g79850 F19K16.19	100	149	0	0	1	149	1	149	1.4E-103	302
AT2G04400.1	sp|P49572|TRPC_ARATH	reviewed	Indole-3-glycerol phosphate synthase, chloroplastic (IGPS) (EC 4.1.1.48)	IGPS At2g04400 T1O3.19	100	402	0	0	1	402	1	402	0	820
AT2G05580.1	tr|Q9SL09|Q9SL09_ARATH	unreviewed	Glycine-rich protein family	At2g05580 T20G20.7 T20G20_7	100	302	0	0	1	302	1	302	1.2E-153	441
AT2G13360.1	sp|Q56YA5|SGAT_ARATH	reviewed	Serine--glyoxylate aminotransferase (EC 2.6.1.45) (Alanine--glyoxylate aminotransferase) (AGT) (EC 2.6.1.44) (Asparagine aminotransferase) (EC 2.6.1.-) (Serine--pyruvate aminotransferase) (EC 2.6.1.51)	AGT1 At2g13360 F14O4.7	100	401	0	0	1	401	1	401	0	828
AT2G21660.1	sp|Q03250|RBG7_ARATH	reviewed	Glycine-rich RNA-binding protein 7 (AtGR-RBP7) (AtRBG7) (Glycine-rich protein 7) (AtGRP7) (Protein COLD, CIRCADIAN RHYTHM, AND RNA BINDING 2) (Protein CCR2)	RBG7 CCR2 GR-RBP7 GRP7 At2g21660 F2G1.7	100	176	0	0	1	176	1	176	2.6E-104	306
AT2G24200.1	sp|P30184|AMPL1_ARATH	reviewed	Leucine aminopeptidase 1 (EC 3.4.11.1) (Leucyl aminopeptidase 1) (AtLAP1) (Proline aminopeptidase 1) (EC 3.4.11.5) (Prolyl aminopeptidase 1)	LAP1 PM25 At2g24200 F27D4.11	100	520	0	0	1	520	1	520	0	1052
AT2G29350.1	sp|Q9ZW18|SAG13_ARATH	reviewed	Senescence-associated protein 13 (Tropinone reductase homolog SAG13) (EC 1.1.1.-)	SAG13 At2g29350 F16P2.27	100	269	0	0	1	269	1	269	0	557
AT2G30870.1	sp|P42761|GSTFA_ARATH	reviewed	Glutathione S-transferase F10 (AtGSTF10) (EC 2.5.1.18) (AtGSTF4) (GST class-phi member 10) (Protein EARLY RESPONSE TO DEHYDRATION 13)	GSTF10 ERD13 GSTF4 At2g30870 F7F1.8	100	215	0	0	1	215	1	215	1.3E-154	437
AT2G41100.1	sp|P25071|CML12_ARATH	reviewed	Calmodulin-like protein 12 (Touch-induced calmodulin-related protein 3)	CML12 CAL4 TCH3 At2g41100 T3K9.13	100	324	0	0	1	324	1	324	0	666
AT2G43560.1	sp|O22870|FK163_ARATH	reviewed	Peptidyl-prolyl cis-trans isomerase FKBP16-3, chloroplastic (PPIase FKBP16-3) (EC 5.2.1.8) (FK506-binding protein 16-3) (AtFKBP16-3) (Immunophilin FKBP16-3) (Rotamase)	FKBP16-3 FKBP17 At2g43560 T1O24.30	100	223	0	0	1	223	1	223	4.4E-156	441
AT2G45220.1	sp|O22149|PME17_ARATH	reviewed	Probable pectinesterase/pectinesterase inhibitor 17 [Includes: Pectinesterase inhibitor 17 (Pectin methylesterase inhibitor 17); Pectinesterase 17 (PE 17) (EC 3.1.1.11) (Pectin methylesterase 17) (AtPME17)]	PME17 ARATH17 At2g45220 F4L23.27	100	511	0	0	1	511	1	511	0	1057
AT3G07390.1	sp|Q94BT2|AIR12_ARATH	reviewed	Auxin-induced in root cultures protein 12	AIR12 At3g07390 F21O3_10	100	252	0	0	22	273	1	252	2.3E-178	501
AT3G09260.1	sp|Q9SR37|BGL23_ARATH	reviewed	Beta-glucosidase 23 (AtBGLU23) (EC 3.2.1.21) (Protein PHOSPHATE STARVATION-RESPONSE 3.1)	BGLU23 PSR3.1 PYK10 At3g09260 F3L24.13	100	524	0	0	1	524	1	524	0	1098

AT3G15356.1	sp|Q9LJR2|LECT2_ARATH	reviewed	Lectin-like protein LEC (AtLEC) (Ath.lec2)	LEC At3g15356 K7L4.17	100	271	0	0	1	271	1	271	0	553
AT3G16420.1	sp|O04314|JAL30_ARATH	reviewed	PYK10-binding protein 1 (Jacalin-related lectin 30) (Jasmonic acid-induced protein)	PBP1 JAL30 JIP PBPI At3g16420 MDC8.5 T02O04.8	100	298	0	0	1	298	1	298	0	603
AT3G16460.1	sp|O04310|JAL34_ARATH	reviewed	Jacalin-related lectin 34	JAL34 At3g16460 T02O04.4	100	705	0	0	1	705	1	705	0	1363
AT3G28270.2	sp|Q9LHD9|U496C_ARATH	reviewed	UPF0496 protein At3g28270	At3g28270 MZF16.5	100	374	0	0	1	374	1	374	0	752
AT3G44300.1	tr|Q1LYZ1|Q1LYZ1_ARATH	unreviewed	At3g44300 (Nitrilase 2)	At3g44300	100	339	0	0	1	339	1	339	0	705
AT3G47070.1	tr|Q9SD66|Q9SD66_ARATH	unreviewed	Thylakoid soluble phosphoprotein	F13I12.120 At3g47070	100	100	0	0	1	100	1	100	6.08E-64	197
AT3G49120.1	sp|Q9SMU8|PER34_ARATH	reviewed	Peroxidase 34 (Atperox P34) (EC 1.11.1.7) (ATPCb)	PER34 P34 PRXCB At3g49120 F2K15.3 T2J13.40	100	353	0	0	1	353	1	353	0	726
AT3G54640.1	sp|Q42529|TRPA2_ARATH	reviewed	Tryptophan synthase alpha chain, chloroplastic (EC 4.2.1.20) (Indole-3-glycerol-phosphate lyase, chloroplastic) (EC 4.1.2.8) (Protein TRYPTOPHAN-REQUIRING 3)	TSA1 TRP3 TSA2 At3g54640 T14E10.210	100	312	0	0	1	312	1	312	0	623
AT3G60900.1	sp|Q9LZX4|FLA10_ARATH	reviewed	Fasciclin-like arabinogalactan protein 10	FLA10 At3g60900 T4C21.310	100	422	0	0	1	422	1	422	0	828
AT3G61440.1	sp|Q9S757|CYSC1_ARATH	reviewed	Bifunctional L-3-cyanoalanine synthase/cysteine synthase C1, mitochondrial (EC 2.5.1.47) (EC 4.4.1.9) (Beta-substituted Ala synthase 3;1) (ARAth-Bsas3;1) (Cysteine synthase C1) (AtCYSC1) (O-acetylserine (thiol)-lyase 5)	CYSC1 OAS5 At3g61440 F2A19.40	100	368	0	0	1	368	1	368	0	756
AT3G63140.1	sp|Q9LYA9|CP41A_ARATH	reviewed	Chloroplast stem-loop binding protein of 41 kDa a, chloroplastic (CSP41-a)	CSP41A At3g63140 T20O10.240	100	406	0	0	1	406	1	406	0	828
AT4G02520.1	sp|P46422|GSTF2_ARATH	reviewed	Glutathione S-transferase F2 (AtGSTF2) (EC 2.5.1.18) (24 kDa auxin-binding protein) (AtPM24) (GST class-phi member 2)	GSTF2 PM24.1 At4g02520 T10P11.18	100	212	0	0	1	212	1	212	1.3E-154	436
AT4G03280.1	sp|Q9ZR03|UCRIA_ARATH	reviewed	Cytochrome b6-f complex iron-sulfur subunit, chloroplastic (EC 7.1.1.6) (Plastohydroquinone:plastocyanin oxidoreductase iron-sulfur protein) (Proton gradient regulation protein 1) (Rieske iron-sulfur protein) (ISP) (RISP)	petC PGR1 At4g03280 F4C21.21	100	229	0	0	1	229	1	229	2.3E-168	473
AT4G05180.1	sp|Q41932|PSBQ2_ARATH	reviewed	Oxygen-evolving enhancer protein 3-2, chloroplastic (OEE3) (16 kDa subunit of oxygen evolving system of photosystem II) (OEC 16 kDa subunit)	PSBQ2 PSBQB At4g05180 C17L7.100	100	230	0	0	1	230	1	230	2E-163	460
AT4G08770.1	sp|Q9LDN9|PER37_ARATH	reviewed	Peroxidase 37 (Atperox P37) (EC 1.11.1.7) (ATP38)	PER37 P37 At4g08770 T32A17.80	100	346	0	0	1	346	1	346	0	711
AT4G15530.5	sp|O23404|PPDK1_ARATH	reviewed	Pyruvate, phosphate dikinase 1, chloroplastic (EC 2.7.9.1) (Pyruvate, orthophosphate dikinase 1)	PPDK At4g15530 dl3805c FCAALL.325	100	963	0	0	1	963	1	963	0	1982
AT4G16260.1	sp|Q8VZJ2|BGNEM_ARATH	reviewed	Probable glucan endo-1,3-beta-glucosidase At4g16260 (EC 3.2.1.39)	At4g16260 dl4170c	100	344	0	0	1	344	1	344	0	693

AT4G19880.2	tr|F4JU03|F4JU03_ARATH	unreviewed	Glutathione S-transferase family protein	At4g19880 T16H5.240 T16H5_240	100	382	0	0	1	382	1	382	0	803
AT4G23100.1	sp|P46309|GSH1_ARATH	reviewed	Glutamate--cysteine ligase, chloroplastic (EC 6.3.2.2) (Gamma-ECS) (GCS) (Gamma-glutamylcysteine synthetase) (Protein ROOT MERISTEMLESS 1) (AtGCL) (Protein cadmium-sensitive 2) (Protein phytoalexin-deficient 2)	GSH1 CAD2 GCL PAD2 RML1 At4g23100 F7H19.290	100	522	0	0	1	522	1	522	0	1083
AT4G23670.1	tr|Q9SUR0|Q9SUR0_ARATH	unreviewed	AT4G23670 protein (AT4g23670/F9D16_140) (Polyketide cyclase/dehydrase and lipid transport superfamily protein) (Putative major latex protein)	At4g23670 F9D16.140 F9D16_140	100	151	0	0	1	151	1	151	6.1E-108	313
AT4G23680.1	tr|Q9SUQ9|Q9SUQ9_ARATH	unreviewed	AT4g23680/F9D16_150 (Polyketide cyclase/dehydrase and lipid transport superfamily protein) (Putative major latex protein)	At4g23680 F9D16.150 F9D16_150	100	151	0	0	1	151	1	151	1E-108	315
AT4G23710.1	tr|Q0WT72|Q0WT72_ARATH	unreviewed	V-type proton ATPase subunit G	At4g23710	100	106	0	0	1	106	1	106	4.18E-68	208
AT4G27070.1	tr|Q0WS13|Q0WS13_ARATH	unreviewed	Tryptophan synthase (EC 4.2.1.20)	At4g27070	100	475	0	0	1	475	1	475	0	979
AT4G30530.1	sp|Q9M0A7|GGP1_ARATH	reviewed	Gamma-glutamyl peptidase 1 (EC 3.4.19.16)	GGP1 At4g30530	100	250	0	0	1	250	1	250	0	516
AT4G34050.1	tr|A0A1P8B3H0|A0A1P8B3H0_AR	unreviewed	S-adenosyl-L-methionine-dependent methyltransferases superfamily protein	CCoAOMT1 At4g34050 F28A23.190 F28A23_190	100	259	0	0	1	259	28	286	0	539
AT4G34180.1	tr|Q93V74|Q93V74_ARATH	reviewed	Cyclase-like protein 1	CYCLASE1 At4g34180 F10M10.6	100	255	0	0	1	255	1	255	0	523
AT4G34200.1	sp|O49485|SERA1_ARATH	reviewed	D-3-phosphoglycerate dehydrogenase 1, chloroplastic (EC 1.1.1.95) (Protein EMBRYO SAC DEVELOPMENT ARREST 9)	PGDH1 EDA9 At4g34200 F10M10.7	100	603	0	0	1	603	1	603	0	1208
AT4G35630.1	sp|Q96255|SERB1_ARATH	reviewed	Phosphoserine aminotransferase 1, chloroplastic (AtPSAT1) (EC 2.6.1.52) (Phosphohydroxythreonine aminotransferase)	PSAT1 At4g35630 F8D20.140	100	430	0	0	1	430	1	430	0	893
AT4G37520.1	sp|Q43731|PER50_ARATH	reviewed	Peroxidase 50 (Atperox P50) (EC 1.11.1.7) (ATP9a) (PRXR2)	PER50 P50 At4g37520 F19F18.10 F6G17.9	100	329	0	0	1	329	1	329	0	678
AT5G02960.1	tr|D7M7Y0|D7M7Y0_ARALL	unreviewed	40S ribosomal protein S23	ARALYDRAFT_487072	100	142	0	0	1	142	1	142	1.61E-97	286
AT5G12030.1	sp|O81822|HS177_ARATH	reviewed	17.7 kDa class II heat shock protein (17.7 kDa heat shock protein) (AtHsp17.7)	HSP17.7 At5g12030 F14F18.200	100	156	0	0	1	156	1	156	1.1E-109	318
AT5G14910.1	tr|Q93VK7|Q93VK7_ARATH	unreviewed	AT5g14910/F2G14_30 (Heavy metal transport/detoxification superfamily protein)	At5g14910 F2G14.30 F2G14_30	100	178	0	0	1	178	1	178	1.4E-125	360
AT5G19940.1	sp|Q941D3|PAP8_ARATH	reviewed	Probable plastid-lipid-associated protein 8, chloroplastic (Fibrillin-6)	PAP8 FBN6 FIB6 At5g19940 F28I16_90	100	239	0	0	1	239	1	239	1.2E-172	484
AT5G47190.1	sp|Q8RXX5|RK192_ARATH	reviewed	50S ribosomal protein L19-2, chloroplastic	At5g47190 MQL5.4	100	229	0	0	1	229	1	229	1.8E-162	457

AT5G66760.1	sp|O82663|SDHA1_ARATH	reviewed	Succinate dehydrogenase [ubiquinone] flavoprotein subunit 1, mitochondrial (EC 1.3.5.1) (Flavoprotein subunit 1 of complex II) (FP)	SDH1-1 At5g66760 MSN2.16	100	634	0	0	1	634	1	634	0	1324
ATCG00540.1	tr|A0A1B1W4V8|A0A1B1W4V8_A	unreviewed	Cytochrome f	petA	100	320	0	0	1	320	1	320	0	648
AT3G47800.1	tr|Q9STT3|Q9STT3_ARATH	unreviewed	Aldose 1-epimerase (EC 5.1.3.3) (Galactose mutarotase)	T23J7.130 At3g47800	100	358	0	0	1	358	1	358	0	737
ATCG00770.1	tr|A0A249RSI7|A0A249RSI7_RAPSA	unreviewed	30S ribosomal protein S8, chloroplastic	rps8	100	134	0	0	1	134	1	134	1.6E-89	265

Supplementary Table S4. Top-10 entries deriving from functional enrichment analysis of differentially represented proteins in leaves from WT and *bzip28 bzip60* mutant *Arabidopsis* plants infected by *D. gigantea* for 24 h. Results derive from an independent pairwise comparison of leaves of infected WT *vs* non-infected WT plants as well as of infected *bzip28 bzip60* mutant *vs* non-infected *bzip28 bzip60* mutant plants, which were further analyzed for Biological Process (GO) are shown.

Biological Process (GO)			
GO-term	***Description***	***Count in gene set***	***False discovery rate***
GO:0050896	Response to stimulus	39 of 5064	1.44e-09
GO:0042221	Response to chemical	29 of 2654	1.44e-09
GO:0009636	Response to toxic substance	13 of 330	1.44e-09
GO:0046686	Response to cadmium ion	12 of 286	1.89e-09
GO:0009628	Response to abiotic stimulus	23 of 1699	1.90e-09
GO:0098754	Detoxification	11 of 230	2.22e-09
GO:0042430	Indole-containing compound metabolic process	8 of 69	2.22e-09
GO:0006950	Response to stress	29 of 2932	2.22e-09
GO:0010038	Response to metal ion	13 of 414	3.51e-09
GO:0010035	Response to inorganic substance	16 of 795	8.91e-09

Supplementary Table S5. Top-10 entries deriving from functional enrichment analysis of differentially represented proteins in leaves from WT and *bzip28 bzip60* mutant *Arabidopsis* plants infected by *D. gigantea* for 24 h. Results derive from an independent pairwise comparison of leaves of infected WT *vs* non-infected WT plants as well as of infected *bzip28 bzip60* mutant *vs* non-infected *bzip28 bzip60* mutant plants, which were further analyzed for Molecular Function (GO).

Molecular Function (GO)			
GO-term	***Description***	***Count in gene set***	***False discovery rate***
GO:0048037	Cofactor binding	18 of 860	7.51e-10
GO:0005507	Copper ion binding	9 of 157	3.56e-08
GO:0046872	Metal ion binding	26 of 2940	2.89e-07
GO:0046914	Transition metal ion binding	15 of 933	4.13e-07
GO:0003824	Catalytic activity	40 of 7239	1.12e-06
GO:0016491	Oxidoreductase activity	16 of 1201	1.18e-06
GO:0043295	Glutathione binding	4 of 11	1.21e-06
GO:0050897	Cobalt ion binding	5 of 42	2.42e-06
GO:0043167	Ion binding	31 of 5070	7.64e-06
GO:0004364	Glutathione transferase activity	5 of 55	7.64e-06

Supplementary Table S6. Bridged and non-linked nodes identified during STRING analysis of differentially represented proteins in leaves from WT and *bzip28 bzip60* mutant *Arabidopsis* plants infected by *D. gigantea* for 24 h. Results derive from an independent pairwise comparison of leaves of infected WT *vs* non-infected WT plants as well as of infected *bzip28 bzip60* mutant *vs* non-infected *bzip28 bzip60* mutant plants.

STRING code	***TAIR accession***	***Protein name***
GL22	AT1G02335	Germin-like protein subfamily 2 member 2 precursor
GSTF7	AT1G02920	Glutathione S-transferase F7
GSTF6	AT1G02930	Glutathione S-transferase F6
AT1G16850	AT1G16880	Uncharacterized protein At1g16850
PGDH	AT1G17745	D-3-phosphoglycerate dehydrogenase 2, chloroplastic
BTF3	AT1G17880	Basic transcription factor 3
F5M15.5	AT1G20620	Catalase-3
IAR4	AT1G24180	Thiamin diphosphate-binding fold (THDP-binding) superfamily protein
AT1G26410	AT1G26410	FAD-binding Berberine family protein
TRX5	AT1G45145	Thioredoxin H-type 5
AT1G54010	AT1G54010	GDSL-like Lipase/Acylhydrolase superfamily protein
CRT1a	AT1G56340	Calreticulin 1a
EI305	AT1G62380	1-Aminocyclopropane-1-carboxylate oxidase 2
MLP43	AT1G70890	MLP-like protein 43 (MLP43)
KTI1	AT1G73260	Kunitz trypsin inhibitor 1
OPR1	AT1G76680	12-Oxophytodienoate reductase 1
GSTU20	AT1G78370	Glutathione S-transferase TAU 20
AT1G78830	AT1G78830	Curculin-like (mannose-binding) lectin family protein
RPS17	AT1G79850	30S ribosomal protein S17, chloroplastic
AT2G04400	AT2G04400	Indole-3-glycerol phosphate synthase, chloroplastic
AT2G05580	AT2G05580	Uncharacterized protein At2g05580
AGT	AT2G13360	Serine-glyoxylate aminotransferase
GRP7	AT2G21660	Cold, circadian rhythm, and rna binding 2
LAP1	AT2G24200	Cytosol aminopeptidase family protein
SAG13	AT2G29350	Senescence-associated protein 13
GSTF10	AT2G30870	Glutathione S-transferase PHI 10
TCH3	AT2G41100	Calcium-binding EF hand family protein
AT2G43560	AT2G43560	FKBP-like peptidyl-prolyl cis-trans isomerase family protein
AT2G45220	AT2G45220	Plant invertase/pectin methylesterase inhibitor superfamily
AIR12	AT3G07390	Auxin-induced in root cultures protein 12
PYK10	AT3G09260	Glycosyl hydrolase superfamily protein
AT3G15356	AT3G15356	Legume lectin family protein
JIP	AT3G16420	PYK10-binding protein 1
JAL34	AT3G16460	Mannose-binding lectin superfamily protein
AT3G28270	AT3G28270	Protein of unknown function (DUF677)
NIT2	AT3G44300	Nitrilase 2
AT3G47070	AT3G47070	Uncharacterized protein F13I12.120
PRXCB	AT3G49120	Peroxidase 34
TSA2	AT3G54640	Tryptophan synthase alpha chain, chloroplastic
FLA10	AT3G60900	FASCICLIN-like arabinogalactan-protein 10

CYSC1	AT3G61440	Bifunctional L-3-cyanoalanine synthase/cysteine synthase C1, mitochondrial
CSP41A	AT3G63140	Chloroplast stem-loop binding protein of 41 kDa
GSTF2	AT4G02520	Glutathione S-transferase PHI 2
PETC	AT4G03280	Cytochrome b6-f complex iron-sulfur subunit, chloroplastic
PSBQ-2	AT4G05180	Oxygen-evolving enhancer protein 3-2, chloroplastic
Prx37	AT4G08770	Peroxidase superfamily protein
PPDK	AT4G15530	Encodes a dual-targeted protein believed to act as a pyruvate, orthophosphate dikinase
AT4G16260	AT4G16260	Probable glucan endo-1,3-beta-glucosidase At4g16260
AT4G19880	AT4G19880	Glutathione S-transferase family protein
GSH1	AT4G23100	Glutamate-cysteine ligase, chloroplastic
AT4G23670	AT4G23670	Polyketide cyclase/dehydrase and lipid transport superfamily protein
AT4G23680	AT4G23680	Polyketide cyclase/dehydrase and lipid transport superfamily protein
VAG2	AT4G23710	Vacuolar ATP synthase subunit G2
TSB2	AT4G27070	Tryptophan synthase beta chain 2, chloroplastic
GGP1	AT4G30530	Class I glutamine amidotransferase-like superfamily protein
CCoAOMT1	AT4G34050	S-adenosyl-L-methionine-dependent methyltransferases superfamily protein
AT4G34180	AT4G34180	Cyclase family protein
EDA9	AT4G34200	D-3-phosphoglycerate dehydrogenase 1
PSAT	AT4G35630	Phosphoserine aminotransferase 1, chloroplastic
AT4G37520	AT4G37520	Peroxidase superfamily protein
AT5G02960	AT5G02960	Ribosomal protein S12/S23 family protein
HSP17.6A	AT5G12030	17.7 kDa class II heat shock protein
AT5G14910	AT5G14910	Heavy metal transport/detoxification superfamily protein
AT5G19940	AT5G19940	Plastid-lipid associated protein PAP / fibrillin family protein
AT5G47190	AT5G47190	50S ribosomal protein L19-2, chloroplastic
SDH1-1	AT5G66760	Succinate dehydrogenase [ubiquinone] flavoprotein subunit 1, mitochondrial
PETA	ATCG00540	Photosynthetic electron transfer A
AT3G47800	AT3G47800	Galactose mutarotase-like superfamily protein
RPS8	ATCG00770	30S ribosomal protein S8, chloroplastic
